# Large scale model lipid membrane movement induced by a cation switch

**DOI:** 10.1016/j.jcis.2021.03.078

**Published:** 2021-08-15

**Authors:** Laura H. John, Gail M. Preston, Mark S.P. Sansom, Luke A. Clifton

**Affiliations:** a Department of Biochemistry, University of Oxford, South Parks Road, Oxford OX1 3QU, UK; b ISIS Pulsed Neutron and Muon Source, Science and Technology Facilities Council, Rutherford Appleton Laboratory, Harwell Science and Innovation Campus, Didcot, Oxfordshire OX11 OQX, UK; c Department of Plant Sciences, University of Oxford, South Parks Road, Oxford OX1 3RB, UK

**Keywords:** Biological membranes, Biosensors, Distance tuning, Biomimetic, Model membranes, Cation switch, Calcium, Electrostatics, Neutron reflectometry, Molecular dynamics, Self-assembled monolayer, Cation binding

## Abstract

A biomembrane sample system where millimolar changes of cations induce reversible large scale (≥ 200 Å) changes in the membrane-to-surface distance is described. The system composes of a free-floating bilayer, formed adjacent to a self-assembled monolayer (SAM). To examine the membrane movements, differently charged floating bilayers in the presence and absence of Ca^2+^ and Na^+^, respectively, were examined using neutron reflectivity and quartz crystal microbalance measurements, alongside molecular dynamics simulations. In neutron reflectivity the variation of Ca^2+^ and Na^+^ concentration enabled precision manipulation of the membrane-to-surface distance. Simulations suggest that Ca^2+^ ions bridge between SAM and bilayer whereas the more diffuse binding of Na^+^, especially to bilayers, is unable to fully overcome the repulsion between anionic floating bilayer and anionic SAM. Reproduced neutron reflectivity results with quartz crystal microbalance demonstrate the potential of this easily producible sample system to become a standard analysis tool for e.g. investigating membrane binding effects, endocytosis and cell signaling.

## Graphical abstract

**Figure:**
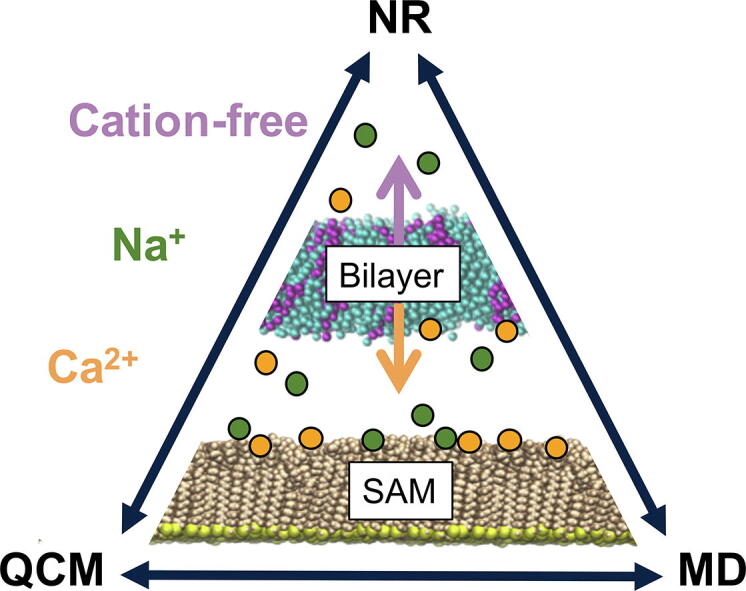

## 1 Introduction

Comprised predominantly of a lipid bilayer with embedded and bound proteins from a large variety of classes, biological membranes are the key structural material of life at the cellular level. Membranes define the external limit of each cell and, within eukaryotic cells, the many organelles, which carry out a multiplicity of cellular functions. Membranes control molecular transport, both into and out of the cell. Therefore, membrane associated proteins are involved in almost all biochemical pathways and account for around ~ 40% of drug targets [1]. Studying membranes *in vivo* is challenging due to their complexity and small transverse size. Model membranes have therefore been key in gaining a molecular level understanding of membrane biochemistry [2], [3]. These systems allow for precision structural and biophysical studies to occur on systems of reduced (whilst defined) complexity compared to those found *in vivo*.

Planar supported lipid membrane samples at interfaces offer an ideal model membrane system due to the variety of benchtop techniques suitable for analysing this sample type [4] and the relative ease of fabrication of these samples by vesicle rupture [5], solvent exchange [6] or monolayer transfer techniques [7]. The simplest models consist of bilayers deposited directly onto a solid support material such as silicon, glass or mica [8], [9]. More advanced forms of planar membrane models use soft polymer supports [10] or surface tethers [11] to reduce surface influence and thus have water on both sides of the membrane model. Floating supported bilayers achieve this through a combination of repulsive and attractive forces between the membrane and the surface [12], [13], [14]. This effect causes the membrane to float ~ 2 nm away from a bulk interface. Consequently, a layer of ‘bulk’ solution is present on both sides of the planar membrane without the need for either tethering or interacting with surface bound material. Recently we discovered that membranes which float 1–3 nm away from a solid interface can be self-assembled *via* vesicle rupture adjacent to oligo (ethylene glycol) alkanthiol self-assembled monolayer surfaces (OEG-SAM) [15]. The self-assembly of the free floating bilayer (FFB) onto the support surface significantly reduces the complexity of the FFB generation protocol, thus opening this sample system up to wider utilization. This is in contrast with previous systems which in general required Langmuir-Blodgett/Langmuir Schaefer deposition to form high quality membranes, and therefore required specialist deposition apparatus and expertise [16], [17], [18].

OEG-SAMs have gained significant interest due to their use in anti-fouling surface coatings [19], with this effect related to the repulsive hydration forces resulting from water layers bound to the EG groups of the SAM [20], [21]. The most commonly studied OEG-SAM contains a terminal hydroxyl group and has, according to the “Whiteside” rules of hydrophilicity, no net charge [22], [23], [24]. Here, and in previous studies on FFB systems, we have used carboxyl terminated (COOH–) OEG-SAMs as a support for FFBs. These do not obey these rules due to partial ionization of the SAM terminal carboxyl groups [25]. Charged surfaces are known to bind oppositely charged counter ions at the solid liquid interface, which plays a key role in many technological processes such as water purification [26] and cation exchange chromatography [27]. However, the interactions of metal cations with partly negatively charged COOH-OEG-SAMs and its resulting surface charge modulation have not been studied.

In a previous study, we observed that the presence of 200 mM NaCl in the bulk solution reversibly increased the bilayer-to-SAM distance but could not identify the cause of this effect [15]. Here, we demonstrate a reversible phenomenon where removing and re-adding a calcium chloride (CaCl_2_) concentration one hundred fold smaller than the NaCl concentration previously studied (2 mM vs. 200 mM) induced substantial reversible changes to the bilayer-to-SAM distance on length scales of >20 nm. As electrolytes seem to have a major effect on the behaviour of the FFB system, and given that the self-assembly process of the FFB onto the COOH-OEG-SAM requires CaCl_2_ present in the buffer solution, the interactions of Ca^2+^ and Na^+^, as well as the water density within the system were of particular interest in this study. Therefore, Ca^2+^ or Na^+^ in the buffer solution and FFB lipid compositions with differing levels of biomimetic accuracy were used in the sample systems investigated.

Neutron reflectometry (NR) was used to examine the interfacial membrane constructs, due to its sensitivity as a structural probe for samples buried within complex environments, such as the solid–liquid flow cells used here. This technique is able to structurally resolve complex assemblies of macromolecules through the use of differential protium/deuterium labelling of the interfacial samples and bulk solution [16], [28]. To obtain a molecular level understanding of the membrane movements relative to the surface observed in the NR measurements, multi-scale molecular dynamics (MD) simulations were undertaken to probe cation and water distribution profiles above the SAM and membranes, as well as cation binding energies to the COOH-OEG-SAM and the different lipid bilayer membranes used in the NR experiments. The combination of these two techniques allowed for a detailed understanding of the ion induced interfacial phenomena. Quartz crystal microbalance (QCM) measurements were additionally utilized to demonstrate the transfer of the bio-mimetic FFB sample system to studies using benchtop analytical techniques.

## 2 Results

### 2.1 NR reveals cation induced bilayer-to-SAM distance modulation

Self-assembled free floating membranes (FFBs) of different lipid compositions adjacent to COOH-OEG-SAMs were generated (Table 1). The complexity of the lipid composition was stepwise increased in order to investigate the feasibility of the system for studies of membrane systems of near-biological complexity. Therefore, starting from a POPC-membrane and a POPC/POPS mixture (8:2, mol:mol), a model of the inner leaflet of the mammalian plasma membrane (POPC/POPS/DOPIP3 7:2:1, mol:mol) [29], [30] and a model approximating the plant plasma membrane (POPC/POPS/Chol/DGDG 5:2:2:1, mol:mol) [31], [32] were produced. As the self-assembling process of FFBs needed 2 mM CaCl_2_ present in the buffer solution and a previous study suggested a significant impact of cations on the bilayer-to-SAM distance, the role of the Ca^2+^ and its interactions within the system were of particular interest. Therefore, each system was treated with 1 mM EDTA to remove Ca^2+^ after the membrane deposition. Then, to investigate the difference between divalent Ca^2+^ and monovalent Na^+^, 200 mM NaCl was added to the system. Finally, the system was again flushed with a 2 mM CaCl_2_ containing solution. NR measurements at three isotopic contrasts (H_2_O, AuMW and D_2_O) were carried out for each system directly after the FFB deposition and after each single treatment. The data fitting quality was high for all the samples studied, which allowed us to resolve the relative distributions of the COOH-OEG-SAM, the membrane, and the water components under all conditions.

**Table 1:** Resolved Key NR Structural Parameters for Studied FFB Samples: The bilayer-to-SAM distance and the bilayer roughness of the different free floating bilayers (FFBs) are given for each applied solution salt condition. Parameter ranges as 95% confidence intervals determined from MCMC resampling of the experimental data fits are given in brackets.

	Bilayer-to-SAM Distance and Bilayer Roughness							
Bilayer	Distance 2 mM CaCl_2_ (initial) [Å]	Roughness 2 mM CaCl_2_ (initial) [Å]	Distance EDTA [Å]	Roughness EDTA [Å]	Distance 200 mM NaCl [Å]	Roughness 200 mM NaCl [Å]	Distance 2 mM CaCl_2_ (final) [Å]	Roughness 2 mM CaCl_2_ (final) [Å]
POPC	13 (11, 16)	5 (4, 7)	201 (195, 207)	63 (60, 65)	38 (36, 40)	9 (7, 10)	13 (11, 15)	3 (0, 5)
POPC:POPS 8:2	12 (10, 13)	8 (7, 9)	594 (576, 611)	125 (113, 138)	100 (93, 107)	55 (51, 59)	11 (9, 13)	6 (4, 8)
POPC:POPS:DOPIP3 7:2:1 (mammalian)	11 (10, 13)	6 (5, 7)	402 (392, 414)	109 (102, 117)	97 (94, 100)	35 (33, 37)	10 (8, 11)	6 (5, 7)
POPC:POPS:Chol:DGDG 5:2:2:1 (plant)	15 (13, 17)	12 (11, 14)	Could not be determined	Could not be determined	121 (118, 125)	31 (30, 34)	14 (12, 16)	10 (8, 11)

**Fig. 1:**
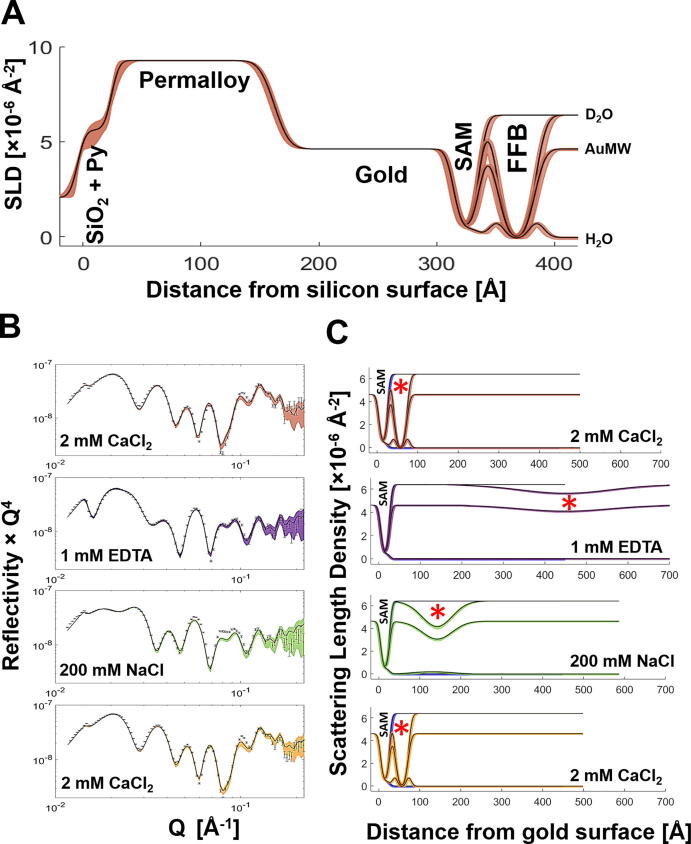
**SLD and Reflectivity Profiles of a Mammalian Plasma Model Membrane in Absence and Presence of Ca^2+^ and Na^+^, respectively: (A)** Full range scattering length density (SLD) profiles of the POPC:POPS:DOPIP3 (7:2:1, mol:mol) free floating bilayer (FFB) adjacent to a COOH-OEG-SAM in the presence of 2 mM CaCl_2_ are shown for the D_2_O, H_2_O and AuMW isotopic contrast. Zero on the x-axis is equal to the silicon/permalloy (Py) interface. **(B, C)** Reflectivity data of the POPC:POPS:DOPIP3 (7:2:1, mol:mol) FFB, including fits are shown for the D_2_O isotopic contrast in **B**. The corresponding SLD profiles are shown in **C**. For clarity, the substrate layers of silicon, permalloy and gold are excluded and zero on the x-axis is equal to the gold/COOH-OEG-SAM interface. Different colours represent different buffer solutions, containing: 2 mM CaCl_2_ (red), 1 mM EDTA (purple), 200 mM NaCl (green) and again added 2 mM CaCl_2_ (orange). The changing position of the bilayer under differing solution salt conditions is shown by a red asterisk (*). (For interpretation of the references to colour in this figure legend, the reader is referred to the web version of this article.)

An example of a full NR data set, including the model data fits and the neutron scattering length density (nSLD) profiles, are shown for the mammalian plasma model membrane (POPC/POPS/DOPIP3 7:2:1, mol:mol) in Fig. 1. For all FFB samples the bilayer-to-SAM distance, together with the FFB roughness, are listed in Table 1. Here, the bilayer-to-SAM distance is defined as that between the COOH-OEG-SAM/water interface and the adjacent bilayer head-group/water interface. Therefore, the bilayer-to-SAM distance is equivalent to the thickness of the water interlayer between the FFB and the COOH-OEG-SAM surface. Further key structural parameters of the NR experiments are listed in Table SI 1. All FFBs showed high bilayer coverage and were in good agreement with previous studies [15], [33].

**Fig. 2:**
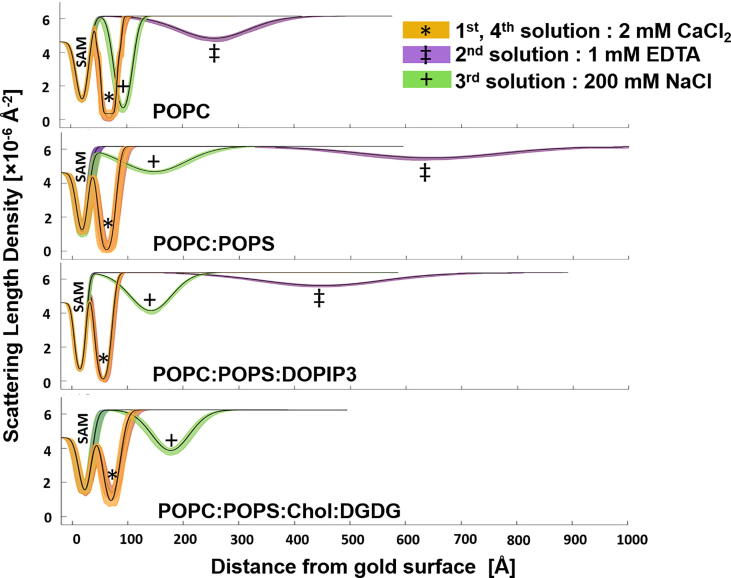
**SLD Profiles of Self-Assembled Free Floating Bilayers (FFBs) at Different Solution Salt Conditions:** Each subplot shows the SLD profiles belonging to one respective membrane (see labels in subplots). Different colours of the profiles represent different additions to the buffer solution, which were present in each sample in following order: 2 mM CaCl_2_ after self-assembling process of the FFB (orange and *), no cations due to 1 mM EDTA mediated Ca^2+^ sequestration (purple and ‡), 200 mM NaCl (green and + ) and finally, again 2 mM CaCl_2_ (orange and *). Changes in the bilayer-to-SAM distance can be seen through changes in the Gaussian distribution of the FFB (here equal to unlabeled distribution in SLDs). The largest changes caused the EDTA mediated Ca^2+^ sequestration, resulting in very rough FFBs, far away from the COOH-OEG-SAM surface. 200 mM NaCl returned the POPC bilayer almost completely, the anionic membranes only partly back towards their initial positions. In presence of negatively charged phospholipids the changes in bilayer-to-SAM distance were larger after Ca^2+^ sequestration (purple) and Na^+^ addition (green), but similar after Ca^2+^ addition (SLD profiles overlaid in orange). Experimental data and model data fits used to produce these SLD profiles are given in supporting information Figures SI 2 to S5. (For interpretation of the references to colour in this figure legend, the reader is referred to the web version of this article.)

All FFB samples showed the same dependency of the bilayer-to-SAM distance and the FFB roughness on the cations present in the buffer solution (see Fig. 2). The initial bilayer-to-SAM distance in the presence of 2 mM CaCl_2_ was at around 10 to 15 Å. EDTA-mediated sequestration of Ca^2+^ resulted in a large increase of the bilayer-to-SAM distance to above 200 Å. The addition of 200 mM NaCl reduced the bilayer-to-SAM distance to an intermediate value, whilst returning the FFB into a 2 mM CaCl_2_ containing buffer solution returned the FFB back to its initial position adjacent to the membrane (~ 10–15 Å). In all cases the reversible movement of the FFB away from and back to the COOH-OEG-SAM surface did not result in measurable loss of the total membrane coverage, suggesting these to be fully reversible processes (see Table SI 1).

Though the trends in bilayer-to-SAM distance change against solution salt conditions were similar, the magnitude differed between FFBs. For example, whilst after Ca^2+^ sequestration the POPC bilayer moved to ~ 200 Å from the COOH-OEG-SAM surface (see Table 1), the anionic eukaryotic membrane models (i.e. POPC:POPS and POPC:POPS:DOPIP3) were found to move to significantly higher distances relative to the COOH-OEG-SAM in this condition (~ 600 and ~ 400 Å respectively, see Table 1). This suggested a correlation between membrane anionic character and the bilayer-to-SAM distance change after Ca^2+^ sequestration. A similar trend was seen with 200 mM NaCl. Here, the POPC sample returned to a close membrane-to-SAM surface distance (~35 Å, compared to ~ 10 Å in 2 mM CaCl_2_) whereas the anionic POPC:POPS and POPC:POPS:DOPIP3 membranes returned to a larger bilayer-to-SAM distance of ~ 100 Å. Interestingly, when comparing the two mammalian anionic membrane models with each other, the bilayer-to-SAM distances during all applied salt solution buffer conditions were greater for the POPC:POPS membrane than for the POPC:POPS:DOPIP3 membrane, although the latter had a higher net negative charge (see Table 1).

The anionic plant plasma membrane model (POPC:POPS:Chol:DGDG, 5:2:2:1, mol:mol) showed the same behaviour as the mammalian plasma membrane models, starting with a close position to the COOH-OEG-SAM in the presence of Ca^2+^ (15 Å (13 Å, 17 Å)), followed by dramatic changes in the reflectivity profile upon EDTA mediated Ca^2+^ sequestration (see data in Figure SI 5). However, the interfacial structure could not be adequately resolved for this cation-free state, most likely due to the roughness of the membrane being too high for analysis or high heterogeneity in the membrane position at this solution condition. Nevertheless, the bilayer-to-SAM distance could be resolved once the sample was immersed into 200 mM NaCl, showing a distance of ~ 120 Å (see Table 1).

In all cases, the bilayer roughness, which describes the height-height correlation function across a layer boundary [15], showed a proportional increase with bilayer-to-SAM distance (see Table 1). In a continuous planar material such as a supported lipid bilayer the largest and therefore overriding source of roughness is likely to be displacement of the bilayer from its mean averaged position due to the fluctuations along the bilayer normal [14], [34]. Therefore, this increase in the roughness value might be related to an increased fluctuation amplitude of the floating membrane as it moves away from the surface [8], [13]. Besides, the increasing bilayer roughness was also proportional to increases in the structural ambiguity of the membrane (see Table 1). This was likely due to membrane SLD profile smearing at high roughness values.

**Fig. 3:**
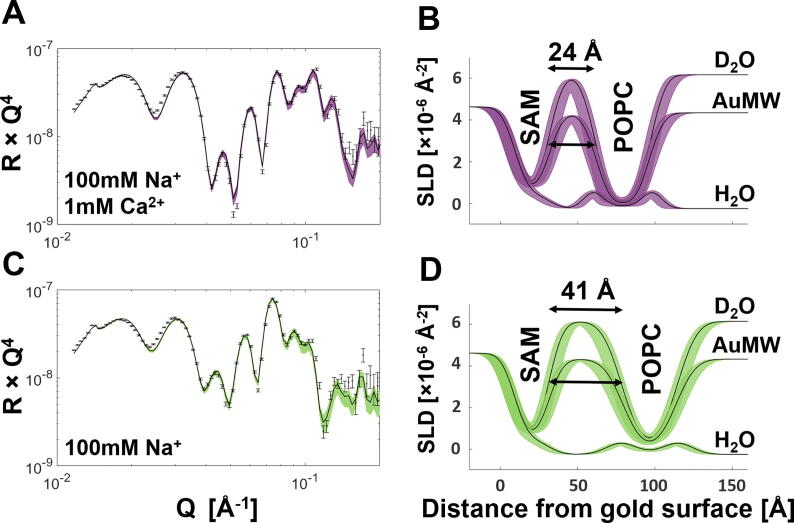
**Ca^2+^ Dependent Fine-tuning of Bilayer-to-SAM Distance in Presence of Na^+^: (A** and **C)** Reflectivity profiles including model data fits, as well as the SLD profiles these fits describe **(B** and **D)** are shown for the D_2_O contrast of a POPC free floating bilayer at two different solution salt conditions: 100 mM NaCl / 1 mM CaCl_2_ (**A** and **B**, purple) and 100 mM NaCl / 0.5 mM EDTA (**C** and **D**, green). The resolved bilayer-to-SAM distances are given in **B** and **D**, highlighting the difference in this caused by 1 mM CaCl_2_ in the presence of 100 mM NaCl. A repeat of these measurements is given in Figure SI 6 and the relevant parameters in Table SI 3. (For interpretation of the references to colour in this figure legend, the reader is referred to the web version of this article.)

Finally, as the NR data suggested that Ca^2+^ brings the FFB much closer to the COOH-OEG-SAM surface than Na^+^, the effect of Ca^2+^ on the bilayer-to-SAM distance in the presence of physiological concentrations of Na^+^ was investigated. To this end, a POPC FFB was sequentially examined in buffer solutions containing 100 mM NaCl with and without 1 mM CaCl_2_. The solution without CaCl_2_ contained 0.5 mM EDTA to remove any contaminating Ca^2+^ cations from the previous buffer solution. The NR analysis (see Fig. 3) revealed that the bilayer-to-SAM distance was significantly smaller with the buffer solution containing both Ca^2+^ and Na^+^ than in the presence of Na^+^ only (~ 23 Å vs*.* ~ 41 Å). These data demonstrate the strong influence of divalent cations on the bilayer-to-SAM distance, even in the presence of a one hundred times higher concentration of monovalent cations. Furthermore, these results suggested the possibility of fine-tuning the bilayer-to-SAM distance by using combinations of mono- and divalent cations. (Figure SI 6 shows an independent repeat of this experiment, confirming the reproducibility of these effects).

### 2.2 Complementary analysis on QCM-D sensor surfaces

Quartz crystal microbalance with dissipation monitoring (QCM-D) is a surface sensitive technique utilizing the piezoelectric effect for real-time measurements of surface interactions. The change in the resonant frequency (Δ*f*) is inversely proportional to the amount of mass at the sensor surface, as given by the Sauerbrey equation [35], [36]. To complement NR measurements and to demonstrate utilization of this sample system in studies using benchtop analytical techniques, FFBs of POPC:POPS (8:2, mol:mol) were self-assembled onto a COOH-OEG-SAM coated QCM sensor chip surface. Measurements of changes in the frequency (Δ*f*) and dissipation (Δ*d*) were used to monitor the sample response after changing the solution salt condition. This is based on the observation that the solution between the FFB and the COOH-OEG-SAM surface is trapped and therefore part of the total layer mass [5]. Hence, the changes in bilayer-to-SAM distance will be associated with a decrease in Δ*f* and an increase in Δ*d*. This assumption is supported by the observation that the water inside a vesicle adsorbing onto a QCM sensor adds to the adsorbed mass measured and its loss can be measured during vesicle rupture [36].

**Fig. 4:**
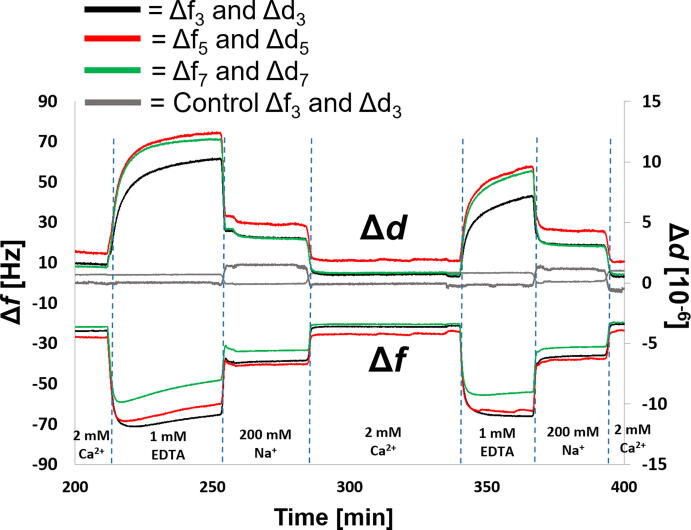
**QCM-D Measurements of a Free Floating POPC:POPS Bilayer (8:2, mol:mol) in Absence and Presence of CaCl_2_ and NaCl, respectively:** The changes in frequency (Δ*f*) and dissipation (Δ*d*) at different solution salt conditions are given for the 3rd (black), 5th (red) and 7th (green) overtones. For the control data set with no membrane adjacent to the COOH-OEG-SAM only the 3rd overtone is given (grey line). Repeated data sets are given in Figure SI 8. (For interpretation of the references to colour in this figure legend, the reader is referred to the web version of this article.)

The QCM-D results were complementary to those observed with NR, showing a large decrease in Δ*f* and increase in Δ*d* when the buffer condition was changed from 2 mM CaCl_2_ to 0 mM CaCl_2_ by EDTA mediated Ca^2+^ sequestration. This implies that a thicker water layer was trapped between the COOH-OEG-SAM and the FFB in case of the Ca^2+^ free state compared to the initial state given in the presence of Ca^2+^ (see Fig. 4). Interestingly, Δ*f* often after an initial decrease showed a slow increase, whereas Δ*d* increased always to a plateau after Ca^2+^ sequestration. It is possible that this effect was due to the movement of the FFB to a large distance, which was outside the frequency depth sensitivity range of QCM-D (which is inversely proportional to square of the resonant frequency) and/or at which the water was no longer mechanically coupled to the FFB. Besides the distance, increased membrane curvature and fluctuation amplitude of the FFB could have been another cause of the reduction in mechanically coupled water to the FFB. Immersing the FFB into 200 mM NaCl containing buffer solution seemed to bring the FFB partly back to the COOH-OEG-SAM, revealed by values of Δ*f* and Δ*d* between that observed in the presence of Ca^2+^ and the cation-free state. Finally, when the samples were exchanged back into 2 mM CaCl_2_ containing buffer solution the Δ*f* and Δ*d* values returned to their initial values. This suggested, like the NR data indicated, a reversible process of FFB-movement away from and back to a close position to the SAM. The solution exchange process was repeated twice for each sample and showed good reversibility, as well as robustness of the biomimetic sample system to repeated large distance changes (see Figure SI 8 for independent repeat and Figure SI 7 for first cycle of these measurements on the same sample systems demonstrating reproducibility between samples as well as within samples).

### 2.3 MD simulations reveal differences in ion binding between COOH-OEG-SAM and membranes

Taken together, the NR and QCM-D results indicated that Ca^2+^ reversibly moves the FFB closest to the COOH-OEG-SAM, followed by Na^+^. These results raised the question what the difference is between Na^+^- and Ca^2+^-interactions within the system. In particular, we wished to understand which layer preferentially interacted with cations, and how strongly the different cationic species interacted. This information would enable us to understand the observed substantive, reversible movements of the FFB away from the surface in response to changes in cation concentrations.

**Fig. 5:**
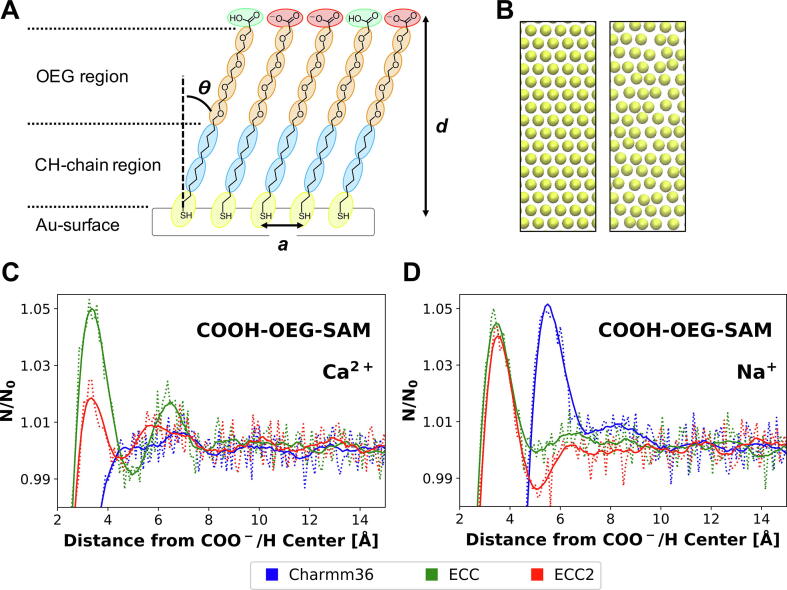
**Structure of COOH-OEG-SAM and Water Distribution Functions Adjacent to an All Atom (AA) Model of COOH-OEG-SAM: (A)** Schematic structure of COOH-OEG-SAM, assembled onto the gold-sputtered surface, including the chemical structure and CG mapping schemes of the SAM molecules. Colours represent different Martini beads. Yellow: SC5, blue: C1, orange: SN0, red: SQn, green: SP2. Calculated parameters like thickness (d), lattice constant (a) and tilt angle (ϴ) of the COOH-OEG-SAM are indicated. **(B)** Bottom view of the initial positioning of the SC5-beads of the SAM-molecules along a hexagonal grid (left), as well as after a 60 ns CG production simulation (right). **(C, D)***N/N_0_* water molecules along surface normal with *N* being the number of molecules at given z-position and *N_0_* the bulk concentration as average of number density above 4 nm away from the surface. **(C)** Results for samples with 400 mM Ca^2+^ and **(D)** with 400 mM Na^+^. Different colours represent three applied Charmm36 force field parameters (see Methods for details): Standard (blue), ECC (green), ECC2 (red). Zero on the x-axis is equal to the centre of mass of the SAM carboxyl groups. (For interpretation of the references to colour in this figure legend, the reader is referred to the web version of this article.)

MD simulations of the COOH-OEG-SAM and of the different lipid bilayers used in the NR experiments were performed, at different CaCl_2_ and NaCl concentrations. Coarse grained (CG) simulations were used to generate initial model structures, using the well-established Martini force field [37], [38]. For the CG model of the COOH-OEG-SAM a range of initial lattice constants (*a_0_*) of the hexagonal COOH-OEG-SAM-grid (0.1 Å intervals between 4.9 and 5.6 Å) were defined for a set of COOH-OEG-SAMs differing in the percentage of charged SAM-COO^–^ molecules present (0, 5, 10, 20, 39 and 50%). Fig. 5 shows a schematic of a partly negatively charged COOH-OEG-SAM. Three replicas each of 60 ns production time for 48 different initial configurations were carried out. The lattice constant *<a>*, tilt angle *<*ϴ*>* of the alkane thiol chain (estimated via order parameters between the thiol-bead and the upper CH-chain-bead *<S_THI-C1B_>* ), the monolayer thickness *<d>* and the surface hydrophilicity *<η>* were monitored to characterize the resultant COOH-OEG-SAM compared to experiments. The averaged results of the last 50 ns of the CG production simulations are shown in Figure SI 11, with the errors representing the standard deviation between the three independent simulations of each system.

Charged SAM-COO^–^ and uncharged SAM-COOH molecules showed negligible differences in order parameters *<S_THI-C1B_>* within all SAM compositions (Figure SI 11, A/B). This explains why *<a>* and *<d>* seemed to be independent of the percentage of charged COOH-OEG-SAM molecules (Figure S11, C/D). Thus, for further simulations the 39% charged SAM was chosen, corresponding to the COOH-OEG-SAM used in NR at 7.2 pH employing the pKa value of carboxyl terminated SAMs predicted by previous studies [25]. However, *<d>* and *<S_THI-C1B_>* showed a high dependency on *a_0_*. Previous studies showed that with the Martini bead definition it is not possible to reproduce the experimental alkane thiol chain tilt angle of 20-35° [39], [40] while matching the experimental lattice constant of 4.97 Å [41] on gold (1 1 1). [42] This was resolved when converting the CG presentation into an all-atom (AA) representation by rescaling the *xy* coordinates by 0.93 ×. A tilt angle of 20-35° corresponds to 0.51–0.82 *<S_THI-C1B_>* which needed *a_0_* to be bigger than 5.1 Å (Figure SI 11, A/B). An initial lattice constant of 5.2–5.3 Å agreed well with the COOH-OEG-SAM thickness observed in NR (20–22 Å, Table S5 and Figure S11, D). The averaged *<a>* showed the overall structural stability of the packing (Figure SI 11, C) and revealed that both *a_0_* of 5.2 and 5.3 Å converged due to minor rearrangements to 5.3 Å. Thus, an *a_0_* of 5.3 Å was chosen for further simulations. The hydrophilicity of the SAM seemed to be almost independent of *a_0_* (Figure SI 11, E).

The CG COOH-OEG-SAM was converted into an AA representation and simulated for up to 100 ns in the presence of different Na^+^ and Ca^2+^ concentrations. To explore the robustness of our results to the parametrization of these ions we explored a number of variations, namely: Charmm36 [43], the electronic continuum correction (ECC) within Charmm36 [44], and a modified ECC version developed using Charmm36, here called ECC2 [45] (see section methods for details). In order to maintain a finite cation concentration in the water phase during all conditions, the cation concentration was raised to 400 mM (see Figure SI 16). As anticipated, we observed oscillations in the water density close to the COOH-OEG-SAM surface, extending approximately four molecular water diameters away from the surface (Fig. 5, C/D), in agreement with previous studies on OEG-SAMs [46], [47].

**Fig. 6:**
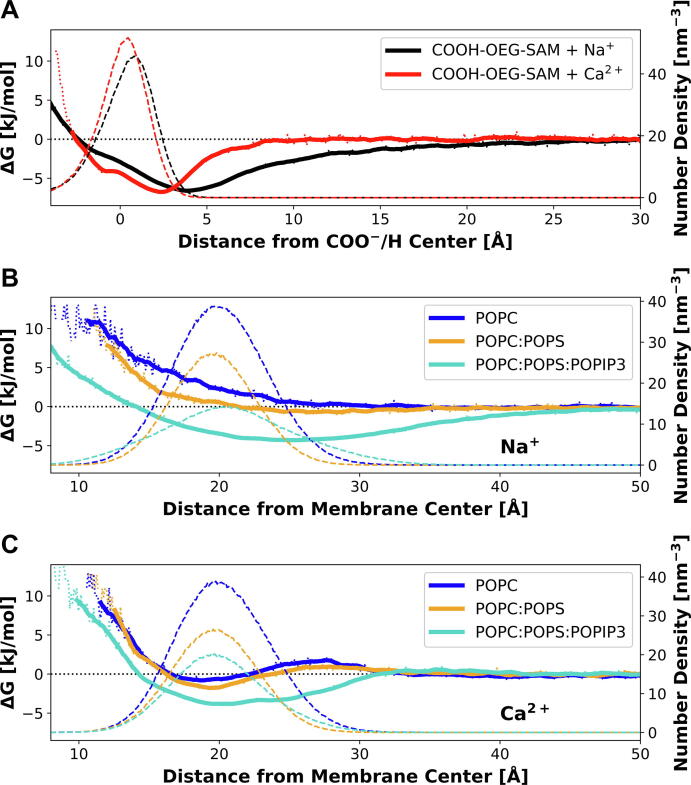
**Free Energy of Ion-Binding in All Atom Simulations of OEG-COOH-SAM and of Lipid Bilayer Membrane**s**: (A)** Free energy profiles for cation interactions with the OEG-COOH-SAM (solid lines). These were evaluated in the presence of 400 mM Ca^2+^ (red) and 400 mM Na^+^ (black) using the ECC2 force field. Zero on the x-axis is equal to the centre of mass of the COOH-OEG-SAM carboxyl groups. Dashed lines represent the corresponding number density profiles of the terminal COO^–^/H groups of the SAM-molecules **(B, C)** Free energy profiles for Na^+^ (B) and Ca^2+^ (C) interactions with phospholipid bilayers (solid lines). The different colours represent the different bilayers: Blue: POPC, orange: POPC:POPS (8:2, mol:mol), cyan: POPC:POPS:POPIP3 (7:2:1, mol:mol). Zero on the x-axis is equal to the centre of mass of the respective bilayer. Dashed lines represent the corresponding number density profiles of the lipid head groups, including the inner phosphates. (For interpretation of the references to colour in this figure legend, the reader is referred to the web version of this article.)

The minima of the free energy landscapes for Na^+^ and for Ca^2+^ ions above the COOH-OEG-SAM were comparable with values of ~ 6.7 and ~ 6.8 kJ/mol (Fig. 6, A), but with closer approach for Ca^2+^ (d_MIN_ = ~ 2.3 Å for Ca^2+^ and ~ 3.9 Å for Na^+^). In each case there was a shoulder at ~ -1 Å suggesting a degree of penetration of cations between the OEG-carboxyl groups (as can be seen from the corresponding density profiles in Figure SI 14). However, for Ca^2+^ the potential well is steeper such that cation density extends for ~ 7 Å from the carboxylates at the surface of the SAM, whereas for Na^+^ the potential well is broader and bulk cation concentration is not reached until > 15 Å from the SAM surface. Comparable results for the standard Charmm36 and the ECC force field parameterization are represented in the SI.

We carried out similar calculations for interactions of the cations with the various lipid bilayer surfaces (Fig. 6, B/C). Two trends are seen: as was observed with the SAM, Ca^2+^ ions are more strongly localized at the surface than are Na^+^ ions, corresponding to clear energy minima near the bilayer head groups and a steeper potential well for Ca^2+^. Furthermore, a clear trend was observed in terms of the depth of the energy well for Ca^2+^ when comparing a zwitterionic POPC bilayer with a modestly anionic POPC/POPS bilayer and with a more markedly anionic POPC/POPS/POPIP3 bilayer. With increasing average surface charge density from 0 e/nm^2^ for the POPC, 0.3 e/nm^2^ for the POPC/POPS to 1.2 e/nm^2^ for the POPC/POPS/POPIP3 bilayer, the depth of the energy well intensified for Ca^2+^. In agreement with this trend, the SAM showed, with a surface charge density of 1.9 e/nm^2^, the deepest energy well for Ca^2+^ compared to the various bilayers. Significant Na^+^ binding was only observed in the presence of the highly negatively charged POPIP3. POPIP3 seemed to cause for Na^+^, as well as for Ca^2+^ an intensified breadth of the energy well, suggesting strongly fluctuating binding (see also Figure SI 15). So, in the case of the POPIP3 containing bilayer, a clear Ca^2+^ ion density extending 10 Å and a clear Na^+^ ion density extending 20 Å from the bilayer surface were observed.

## 3 Discussion

The NR results reveal a clear trend in bilayer-to-SAM distance depending on the salt solution present between the SAM and the lipid bilayer. These trends were similar for a simple POPC and more complex and biologically relevant POPC:POPS, POPC:POPS:DOPIP3, and POPC:POPS:Chol:DGDG free floating bilayers (FFBs). All four samples yielded close approach of the lipid bilayer to the COOH-OEG-SAM surface in the presence of 2 mM CaCl_2_. In the absence of additional cations, a separation of > 200 Å was observed. In the presence of 200 mM NaCl an intermediate separation distance was observed, this being greater for the anionic membranes and less for the zwitterionic POPC bilayer. These results suggest attraction between bilayer and COOH-OEG-SAM is greatest in the presence of Ca^2+^, with a weaker attraction in the presence of an elevated concentration of Na^+^.

The interplay of four main intermolecular forces is thought to control the distance between the FFBs and the COOH-OEG-SAM surface: van der Waals (vdW) forces give rise to weak yet long range attraction between the bilayer and the surface [48], whilst entropic Helfrich fluctuational forces [49], as well as hydration forces can produce a repulsion between the COOH-OEG-SAM and the FFB [13], [50]. Electrostatic forces can produce short range attractive or repulsive interactions depending on the surface charge of the SAM and the FFB [48], [51]. As both the membrane head-groups and the COOH-OEG-SAM surface contained ionizable groups and given the salt solution effects it was inferred that the predominant cause of these effects were changes in electrostatics interactions of the system under differing conditions.

**Fig. 7:**
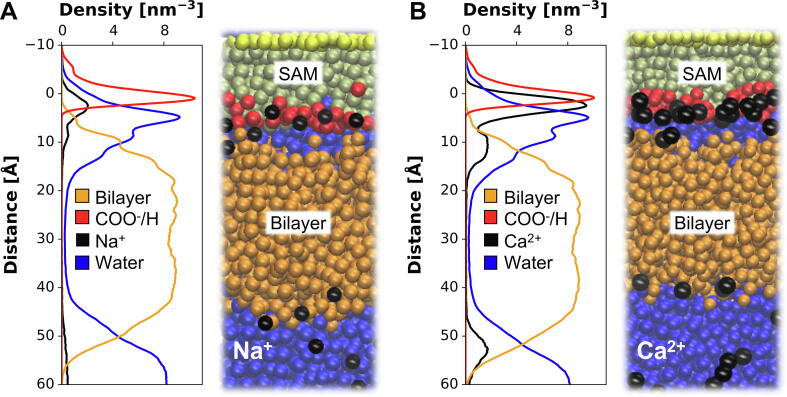
**Coarse Grained Free Floating Bilayer System:** COOH-OEG-SAM and a POPC:POPS (8:2) bilayer were initially separated by a 12 Å water layer in the presence of 400 mM Na^+^**(A)** or Ca^2+^**(B)**, respectively. The bilayer had a hole with a diameter of 2 nm through which ions and water could pass the membrane. After 1 µs production run the number density profiles of the cations and water were determined. Zero on the y-axis is equal to the centre of mass of the COOH-OEG-SAM carboxyl groups. In order to visualize the density profiles of the ions, their density was multiplied by 10. The Ca^2+^ showed a significantly stronger binding to both SAM and bilayer in comparison to Na^+^. The density profile of Ca^2+^**(B, black)** suggests that Ca^2+^ ions bridge between the anionic SAM and the phospholipid surface. However, the week binding of Na^+^, especially to the bilayer **(A, black)**, might cause a too small bridging effect in order to overcome the repulsive force between the anionic SAM and an anionic bilayer. Again, the hydration layers above the SAM are observable in both cases **(A/B, blue)**. (For interpretation of the references to colour in this figure legend, the reader is referred to the web version of this article.)

The MD results allow us to integrate these experimental results with an underlying molecular model of the COOH-OEG-SAM/FFB system (Fig. 7). Simulations showed a layer of increased Ca^2+^ concentration above the SAM surface with a thickness of around 7 Å and above various bilayers with a thickness of 5 to 10 Å (Fig. 6). Therefore, the full width of the aqueous layer between the SAM and the bilayer surfaces observed by NR (10 to 15 Å) will contain an elevated concentration of Ca^2+^. Note that this would accommodate the three to four water layers seen at the SAM surface (Fig. 5). A coarse grained model of this resultant system is shown in Fig. 7, B. From this integrative model it is evident that the Ca^2+^ ions are potentially able to bridge between the anionic SAM (carboxylate) and phospholipid surfaces. It will be of interest to examine the dynamic behaviour of water and Ca^2+^ ions within this nano-confined salt solution layer in more detail by all atom MD simulations. Applying hereby different ion parametrizations, as well as different water models will be of particular interest. In the case of Na^+^ the much weaker binding, especially to the bilayer surfaces suggests a less intensified bridging effect of the interlayer cations, too small to overcome the repulsion force between the SAM and bilayer resulting in the intermediate position of the bilayer (Fig. 7, A). Additional all atom MD simulations comparing the behaviour of Na^+^ with that of Ca^2+^ within the area between SAM and bilayer will be necessary to confirm the predicted molecular model. The comparison of the free energy profiles obtained from such simulations with our current results would allow us to investigate how SAM and membrane influence each other regarding surface behaviour and potential in the presence of different salt conditions.

Previously we demonstrated that changing the NaCl concentration of the solution around a POPC bilayer deposited adjacent to a COOH-OEG-SAM lead to movement of the membrane away from the SAM surface similar to results presented here [15]. In this previous study it was also observed that decreasing the concentration of NaCl increased the membrane-to-SAM surface distance. However, in 0 mM NaCl (no excess Ca^2+^) the membrane-to-SAM surface distance was similar to that observed in the presence of 2 mM Ca^2+^ here. Based on our current findings this suggests a potential persistence or contamination of multivalent cations in these samples. The stringent control of divalent cations employed in the studies described here enabled significant improvements in the reversibility of the membrane distance tuning compared with our initial report of this sample system. Indeed, it was only through this tight control of solution divalent cations that we could gain a precision understanding of the complex forces which control this novel sample system and uncover the elegant mechanism for membrane-to-surface distance control described here.

## 4 Conclusion

Here we demonstrate an easily assembled floating bilayer sample system which shows an, as yet, unseen range (≥ 200 Å) of reversible bilayer movement from the surface as a result of changes in the solution cation concentrations within physiological ranges. MD simulations suggest that cations, especially Ca^2+^ ions electrostatically bridge the anionic membrane and SAM surfaces, while structured hydration layers above the SAM surface prevent contact between the SAM and bilayer. Changing the nature and concentration of cations allows one to modulate this bridging effect. These results suggest that within a complex interplay of forces acting in the floating bilayer sample system, electrostatic interactions can be manipulated to provide a direct means of controlling the membrane-to-surface distance. This “cation switchable” sample system offers a new opportunity to mimic the intracellular and extracellular environment using a planar membrane model system, which are amenable to structural and biophysical analysis, as well as sensing applications. As QCM measurements confirmed the reversible bilayer movement observed with NR, this sample system has the potential to become a standard, benchtop analysis tool investigating *in vitro* a myriad of membrane related biological processes such as translocation and signalling to name a few. One of the biggest advantages of our sample system is the readily fabrication. Having unravelled the uncertainties about forces caused by cations, the importance of the presence of calcium ions during the entire membrane assembly process became clear. Calcium ions enable a sufficient accumulation of lipid vesicles close to the SAM surface. Thus, maintaining 2 mM calcium ions present in the buffer solution enables straightforward production of a free floating bilayer within minutes. Downstream applications of these readily fabricated sample systems could include usage in diagnostic devices containing tailored and controllable membrane models for examining the interactions of therapeutic agents, as well as potential applications in ion sensing.

## 5 Materials and methods

### 5.1 Materials

POPC (1-palmitoyl-2-oleoyl-*sn*-*glycero*-3-phosphocholine, > 99% purity, catalogue and lot number 850457C-500MG-A-209), POPS (1-palmitoyl-2-oleoyl-*sn*-*glycero*-3-phospho-l-serine, > 99% purity, catalogue and lot number 840034C-25MG-B-251), DOPIP3 (1,2-dioleoyl-*sn*-*glycero*-3-phospho-(1′-myo-inositol-3′,4′,5′-trisphosphate), > 99% purity, catalogue and lot number 850156P-500UG-D-014) and DGDG (digalactosyldiacylglycerol, plant origin, > 99% purity, catalogue and lot number 840524P-25MG-A-018) were obtained from Avanti polar lipids (Alabaster, AL, USA) and used without further purification. COOH-OEG-SAM (HS-C_11_-EG_3_-OCH_2_-COOH, >95% purity, catalogue number TH 003-m11.n3-0.2) was obtained from Prochimia surfaces (Gdansk, Poland). D_2_O (Deuterium oxide, > 99.9% purity, catalogue number 151882–1 KG) and Chol (cholesterol, from sheep’s wool, ≥ 92.5% purity, catalogue number HR1533-500MG, lot number LRAB3056), HEPES buffer salts (N-(2-Hydroxyethyl)piperazine-N′-(2-ethanesulfonic acid) sodium salt, ≥ 99.5% purity, catalogue number H3375-500G) were sourced from Sigma-Aldrich (Gillingham, UK). CaCl_2_ (Calcium chloride, 100% purity, catalogue number C/1400/53), NaCl (sodium chloride, ≥ 95% purity, catalogue number S/3160/60) and EDTA (Ethylenediaminetetraacetic, ≥ 95% purity, catalogue number BP120-500) were sourced from Fisher Scientific (Loughborough, UK). Silicon substrates were obtained from Crystran (Poole, UK) as a bespoke request.

### 5.2 Neutron reflectometry (NR) measurements

NR measurements were carried out using the white beam SURF [52] and CRISP [53] reflectometers at the ISIS Spallation Neutron Source, Rutherford Appleton Laboratory (Oxfordshire, UK), which use neutron wavelengths from 0.5 to 7 Å and 0.5 to 6.5 Å, respectively. The reflected intensity was measured at glancing angles of 0.35°, 0.65° and 1.5° for both instruments. Reflectivity was measured as a function of the momentum transfer, Q_z_ (Q_z_ = (4π sin θ)/λ where λ is wavelength and θ is the incident angle). Data were obtained at a nominal resolution (dQ/Q) of 3.5%. The total illuminated sample length was ~ 60 mm on both instruments.

Details of the liquid flow and liquid chromatography setup used in the experiments describe here are described by us in a previous article [54]. Briefly, solid liquid flow cells were placed onto the instrument sample position and connected to instrument controlled HPLC pumps (either a Hitachi L-7100 on SURF or a Knauer Smartline 1000 on CRISP) which control the change of solution isotopic contrast in the flow cell, as well as the change in solution counter ion concentration and type.

#### 5.2.1 Gold and permalloy coating of silicon crystals:

Piranha acid cleaned silicon crystals (50 × 80 × 15 mm) with a polished 80 × 50 mm face (111 orientation, surface roughness (RMS) ~ 3 Å) were sequentially sputter coated with permalloy (Ni_80_Fe_20_) and gold at the NIST centre for Nanoscience and Technology, Gaithersburg, MD, USA, in a Denton Discovery 550 sputtering chamber. Permalloy was used to bind the gold layer to the silicon substrate as its high neutron scattering length density (SLD) benefits the analysis of the NR data [55].

#### 5.2.2 Self-assembled monolayer coating of gold surfaces:

Gold and permalloy coated silicon substrates were cleaned by UV-ozone cleaning followed by washing with ultra-pure water, drying and a second UV-ozone cleaning procedure. Solutions of terminal carboxyl group functionalized (COOH–) OEG-SAMs were prepared by dissolving this material in absolute ethanol (> 99.8% purity) to a concentration of 70 µM. The cleaned substrates were then incubated in this solution for 48 h in a low light environment at room temperature. After this time the substrates were removed from the thiol containing solution and sequentially washed with ethanol and water before drying under nitrogen.

The COOH-OEG-SAM coated substrates were assembled into ISIS facility standard solid/liquid flow cells while completely submerged in water to prevent air entrapment. Images and a description of this process and the cells used can be found in a recent review by us [56]. Once assembled, the cells were stored at 4 °C prior to analysis of the solid/liquid interface by neutron reflectometry.

#### 5.2.3 Surface structure measurements:

Initially measurements of the COOH-OEG-SAM/gold/permalloy coated substrates were then conducted by measuring NR data under two solution isotopic contrast conditions (H_2_O and D_2_O). Upon confirmation of homogenous SAM coated gold surfaces with suitable surface coverage (≥ 95%), free floating bilayer (FFB) fabrication took place.

#### 5.2.4 Free floating bilayer fabrication:

Small unilammelar vesicles (SUVs) were prepared by dissolving phospholipids in CHCl_3_ and evaporating the solvent under a stream of N_2_ to produce a lipid cake which was further dried under vacuum for 12 h. The dry lipid cake was then immersed in the deposition buffer solution (20 mM HEPES, 2 mM CaCl_2_ pH/D 7.2) and bath sonicated to produce a vesicle suspension with a lipid concentration of 1 mg/mL. This suspension was then sonicated with a bath sonicator to produce SUVs. The final sonication step of SUV preparation was always conducted less than 1 h before deposition of the bilayer samples.

The formation of self-assembled floating bilayers by vesicle rupture onto COOH-OEG-SAMs has been described by us previously [15]. Briefly, the concentration of the SUV suspension was reduced to 0.2 mg/mL and injected into the solid/liquid flow cells using pre-washed disposable syringes at room temperature (~ 20 °C). The flow of the solution was reversed after two cell volumes were injected into the solid/liquid cell and ran in reverse for another two cell volumes (~ 6 mL assuming a 3 mL liquid volume in the solid/liquid flow cells). This process was iterated at least twice.

The formation of the bilayers adjacent to the COOH-OEG-SAM coated gold surface was monitored by NR, specifically looking for changes in the Kiessig fringe spacing in the low Q regime (less than 0.05 Å^−1^). Upon bilayer formation a new set of fringe spacings were observed, characterized by a new minimum at ~ 0.03 Å^−1^, which signified the formation of the FFB adjacent to the SAM/water interface. Then the samples were flushed with 20 mM HEPES, 2 mM CaCl_2_ pH/D 7.2 buffer to remove non-surface bound material and heated to 38 °C. Upon confirmation of membrane formation, the vesicle suspension was reinjected, and the sample incubated for ~ 2 h at 38 °C. Finally, buffer solution was flushed through the cells to remove the excess vesicles, followed by the structural examination of the resulting interfacial assemblage by NR.

#### 5.2.5 Isotopic contrast variation strategy:

The samples measured are composed of hydrogenous lipids adjacent to hydrogenous SAMs grafted onto a gold surface. To gain maximum information on the bilayer coverage and position relative to the COOH-OEG-SAM/water interface it was therefore necessary to measure the samples in D_2_O buffer solutions, as this produced a series of layer interfaces, SAM/water-, water/membrane- and membrane/bulk water-interlayer, which had high SLD differences. These interfaces encoded high amplitude fringes in the experimental data and allowed for unambiguous resolution of the interfacial architecture upon data analysis. Indeed, previously we have been able to show that the idealized labelling strategy of a hydrogenous SAM, deuterated water interlayer, hydrogenous membrane and deuterated bulk water allowed for the solution interlayers between the SAMs and floating membranes to be adequately resolved [15].

Therefore, here, for every sample condition the surface assemblage was measured in D_2_O and 75% D_2_O (gold matched water, AuMW) buffer solutions, with both solution contrast conditions being highly sensitive to the overall membrane and SAM structure, as well as the water inter-layer between them. Additionally, a H_2_O solution isotopic contrast was obtained for each experimental condition measured, as under this solution condition the SAM molecules, lipid tails and the water layer are essentially contrast matched, meaning the position of the lipid head-groups can be resolved independently [56]. Due to the large amount of data obtained in this study, only the D_2_O data sets are shown for all samples as these are most sensitive to the membrane-to-SAM distance.

#### 5.2.6 Neutron reflectivity data analysis:

NR data were analysed using the RasCal fitting package (version 2014b, A. Hughes, ISIS Spallation Neutron Source, Rutherford Appleton Laboratory) which employs an optical matrix formalism [57], [58] to fit layer models of the interfacial structure to the experimental reflectivity data. The program is designed to support the simultaneous fitting of multiple sets where parameters such as the layer thickness and roughness (grading of either the bulk interface or interface between layers) are fully or partially fixed across multiple data sets while the scattering length density and derived values such as volume fraction/component coverage can be assumed to vary. The relationships between the SLD, layer thickness and the lipid/SAM area per molecule was defined in the software’s “custom fitting” option.

The model describing the interfacial layer structure between the silicon substrate (super phase) and the bulk water (subphase) consisted of a SiO_2_ layer, a permalloy layer, a gold layer, the SAM, a water interlayer, inner bilayer head-groups, bilayer tails and outer bilayer head-groups. The order of the respective layers was based on prior knowledge of the arrangement of the “under-layers” (SiO_2_, permalloy and gold) on the substrate surface and the arrangement of the organic layers (SAM, water and bilayer) above the gold surface. The thicknesses of the “under layers” were approximately known due to previous use of these substrates. Therefore, their ranges were set around these values based on previous experience. However, for the organic layers their thicknesses and coverages had a minimum parameter range of zero, meaning these layers were not assumed to be present on the sample surface.

The fitting parameters for the metal native SiO_2_, the permalloy and gold under-layers, as well as the SAM were assumed to be the same across all data sets obtained for one membrane sample under varying salt solution conditions. Therefore, to further constrain the fitting of the SAM, gold and permalloy layers, reflectivity data sets were obtained for the SAM/gold/permalloy coated silicon surfaces without the presence of the bilayer at the bulk interface. Each of these SAM only data sets was simultaneously fitted with the data sets, in which the membrane was present above the respective SAM/gold/permalloy coated silicon surface at varying salt solution conditions. Only the presence and position of the membrane was varied between the simultaneously fitted data sets, leading to unique, low ambiguity solutions for the interfacial structures. Note, each membrane data set included three (H_2_O, D_2_O, AuMW) and each SAM only data set two (H_2_O, D_2_O) solution isotopic contrasts, which were also fitted simultaneously. The fitting parameters across the solution isotopic contrasts of each data set were the same.

The organic layers, i.e. the SAM and the bilayer, were fitted by area per molecule, explicitly associated water (with the head-group regions) and coverage. Thereby the coverage represented the volume fraction of the neutron beam probed surface coated with the membrane or SAM respectively. Non-lipid or non-SAM components of these layers were assumed to be defect associated solution. This strategy was useful for the membrane studies described here as it allowed for the linking of the lipid head group and tail parameters such that the molar ratio of these components was the same (as they are part of the same molecule). This approach minimizes the number of free parameters in the model, and it yields useful quantities, such as hydration or area per molecule.

The procedure was previously described in detail by Hughes *et al* [33]. Briefly, in the custom fitting script the relationship between layer thickness, component volume and area per molecule is defined as:

(1) $$\mathrm{Thickness}=\frac{\mathrm{Volume}}{\mathrm{AreaPerMoelcule}}$$

and component volume, scattering length and scattering length density is:

(2) $$SLD\left(\rho \right)=\frac{\sum b}{\mathrm{Volume}}$$

The volume and the scattering lengths (∑b) of each of the interfacial components were known or calculated. The resulting, used SLD values are given in Table SI 5. By fitting the interfacial lipid area per molecule, the layer thicknesses were calculated within the software and used in the matrix calculation to produce the model reflectivity profiles and to fit these to the experimental data.

In neutron reflectivity data fitting, interlayer roughness is often dealt with the Parratt formalism according to the theory of Nevot and Croce [55]. However, this approximation fails when the roughness of a layer approaches the layer thickness. Instead, we constructed SLD profiles using error functions to represent the interlayer roughness, with the error function width set to the Nevot and Croce roughness value. Then, we resampled the SLD profiles at the interfaces into a series of thin unroughened slabs (analogous to partitioning a curve for a midpoint Riemann sum) and calculated the reflectivity from these unroughened layers. This prevented problems with the large roughness values relative to layer thicknesses.

Finally, error estimation was undertaken using RasCal’s Bayesian Error estimation routines [59], [60], with the log-likelihood function described in terms of chi-squared [59]. Marginalized posteriors were obtained using a Delayed Rejection Adaptive Metropolis algorithm [61], [62], and the best fit parameters taken as the distribution maxima; the uncertainties presented here are from the shortest 95% confidence intervals of each distribution. The NR data sets for the 7:2:1 POPC: POPS: DOPIP3 FFB sample and the RasCal custom model used to analyse this are accessible via https://doi.org//10.5281/zenodo.4434613.

### 5.3 Quartz crystal microbalance with dissipation monitoring (QCM-D) measurements

QCM-D was performed using a Q-sense Q4 Analyzer QCM-D system (Gothenburg, Sweden). Sensors were washed with 0.5% sodium dodecyl sulphate solution, ultra-pure water and ethanol before being dried with a stream of N_2_ and UV-ozone cleaned for 25 min. The sensors were then incubated in a 60 µM solution of COOH-OEG-SAM in absolute ethanol (≥ 99.8% purity) [63] for 48 h at room temperature, under low light conditions. The surfaces were washed with ethanol, ultra-pure water, dried with N_2_ and mounted into the temperature controlled QCM flow cells at 20 °C, which were attached to a calibrated peristaltic pump and initially filled with 20 mM HEPES pH 7.2 buffer. A flow rate of 0.25 mL∙min^−1^ was used throughout, unless otherwise stated. Frequency and dissipation changes (Δ*f* and Δ*d*) were monitored using multiple harmonics (n = 3, 5, 7, 9, 11, 13) of the resonant frequency. However, for clarity, data are reported using the 3rd, 5th and 7th harmonics only (3rd only for the control measurements). A baseline was acquired for 5 min into 20 mM HEPES pH 7.2 buffer before the measurement was started. Buffer was exchanged into 20 mM HEPES, 2 mM CaCl_2_ pH 7.2 prior to the injection of a 0.1 mg/ml POPC: POPS 8:2 (mol/mol) vesicle suspension in the same buffer. Vesicles were prepared in the same way as described for neutron reflectometry measurements. Once the Δ*f_3_* (change in frequency of the 3rd harmonic) reached around −70 Hz the surface was washed with 20 mM HEPES, 2 mM CaCl_2_ pH 7.2 buffer to remove excess vesicles leaving a membrane coated sensor surface. A final change in *f_3_* of ~ -25–30 Hz was observed for the formation of the FFB coated gold surface, in good agreement with previous observations on planar membrane systems [36]. One sensor was not incubated in the presence of vesicles but ran through the same changes of solution salt conditions as the membrane containing samples to act as a control in order to assess the frequency and dissipation response to changes in solution conditions. After bilayer formation the changes in frequency and dissipation upon changing the solution conditions buffer between that containing 2 mM CaCl_2_, 1 mM EDTA and 200 mM NaCl were measured. Like with NR measurements, the membranes were first measured in 2 mM CaCl_2_ containing 20 mM HEPES pH 7.2 buffer, then 1 mM EDTA, 200 mM NaCl and finally back into 2 mM CaCl_2_ containing 20 mM HEPES pH 7.2 buffer. The process was repeated on the same samples at least twice to show repeatability. Experiments were repeated in triplicate.

### 5.4 Molecular dynamics (MD) simulations

#### 5.4.1 Coarse grained (CG) simulation protocol:

CG simulations were run using GROMACS 2019.1. [64] and the CG Martini force field, using the open beta 3.0.b.3.2 version [38], [37]. The systems were minimized with the steepest decent method, followed by 10 ps simulation in the canonical (NVT) ensemble and 200 ns in the isothermal-isobaric (NPT) ensemble with V-rescale temperature coupling at 308 K [65]. Semi-isotropic Berendsen pressure coupling was used for the NPT ensemble coupling at 1 bar with a time constant of 4 ps, normal compressibility of 4.5 × 10^-5^ bar^−1^ and lateral compressibility of 0 bar^−1^ for the COOH-OEG-SAM system or of 4.5 × 10^-5^ bar^−1^ for the various bilayer systems [66]. Finally, a 60 ns production simulation was run with V-rescale temperature coupling at 308 K and semi-isotropic Parrinello-Rahman barostat coupling at 1 bar with a time constant of 12 ps, normal compressibility of 3.0 × 10^-4^ bar^−1^ and lateral compressibility of 0 bar^−1^ for the COOH-OEG-SAM system or of 3.0 × 10^4^ bar^−1^ for the various bilayer systems [67]. Van der Waals (vdW) interactions were shifted from 9 to 11 Å and electrostatics were described by the reaction field method, using a potential shift modifier with a cut-off of 11 Å. Bonds were constrained with the LINCS algorithm. The first 10 ns of the production simulation were considered as additional equilibration and therefore not included in the analysis. Data were analysed in VMD [68] or GROMACS [69]. Force field files are accessible via https://doi.org//10.5281/zenodo.4434613.

#### 5.4.2 All atom (AA) simulation protocol:

AA simulations were performed using GROMACS 2019.1 [64]. and three different force fields: standard (i.e. non-scaled) Charmm36 [43], scaled ECC- [44] and ECC2-Charmm36 [45] (for details see below). A force-switch modifier was used for the van der Waals forces and the Particle-Mesh-Ewald method [70] for the electrostatics. Dispersion corrections were turned off. First, 5000 steps of steepest decent minimization were performed. Second, the systems were equilibrated for 10 ps in the NVT ensemble and for 2 ns in the NPT ensemble. Finally, production simulations with 2 fs time step and virtual-sites on the SAM-molecules were run for up to 100 ns. V-rescale temperature coupling [65] at 308 K using a time constant of 0.1 ps and semi-isotropic Parrinello-Rahmen pressure coupling [67] at 1 bar with a time constant of 2 ps, normal compressibility of 4.5 × 10^-5^ bar^−1^ and lateral compressibility of 0 bar^−1^ for the COOH-OEG-SAM system or of 4.5 × 10^-5^ bar^−1^ for the various bilayer systems were applied. For the analysis the final 5 ns of the production simulations were considered. Data were analysed in VMD [68] or GROMACS [69]. Force field files are accessible via https://doi.org//10.5281/zenodo.4434613.

#### 5.4.3 CG simulation setup:

The implementation of the negatively charged COO^–^-SAM and neutral COOH-SAM molecule within the Martini3 force field [38] was achieved by fitting the results from CG MD simulations (bond length and bond angle distributions) to AA MD simulations with the Charmm36 force field [43], [71]. The initial topology for the Charmm36 was generated by using the Charmm-GUI Ligand Reader and Modeler [72]. The provided parameter estimation from 1 μs AA simulations of COO^–^/H-SAM in water (TIP3P) were taken for the CG simulations and refined if necessary (see Figure SI 10). Using standard Martini3 beads the mapping scheme between CG and AA resulted for both molecules in a SC5 bead for the thioethyl tail group, SN0 beads for the C-O-C-groups and C1 beads for each *n*-butyl group. The carboxyl head group was mapped into a SP2 bead for the neutral and into a SQn bead for the negatively charged state (see Fig. 5).

Assembling the CG SAMs was based on an approach of a previous study [42]. Monolayers out of the implemented SAM molecules were built with a modification of the Python tool *insane.py* in a 105 × 105 × 130 Å box [73]. The modifications of the script involved the introduction of the COOH-SAM and COO^-^-SAM matrix, as well as the positioning of the molecules onto a hexagonal lattice with a defined lattice constant. For the representation of the gold surface a flat surface model, used successfully earlier, was applied [42], [74]. Thus, the thioethyl tail beads of the SAM molecules (SC5) were restrained to a plane with a harmonic force constant of 5000 kJ/(mol nm^2^). The percentage of the randomly distributed negatively charged SAM-COO^-^-molecules was either 0, 5, 10, 20, 39 or 50%. Only the required number of counter-ions in form of sodium and no additional salt concentration was added. For each differently charged system the initial lattice constant was varied between 4.9 and 5.9 Å in intervals of 0.1 Å, resulting in 48 different initial system setups. Each of these system setups was built and simulated three times independently.

The lipid bilayers were generated in a 105 × 105 × 200 Å box using the unmodified Python tool *insane.py* [73]. An exception was the membrane containing phosphatidylinositol (3,4,5)-triphosphate (DOPIP3). Here, the Charmm GUI Martini Bilayer Maker was used providing a box of 155 × 155 × 200 Å [73]. Simulations were performed for pure 1-palmitoyl-2-oleoyl-*sn*-*glycero*-3-phosphocholine (POPC), its mixture with 1-palmitoyl-2-oleoyl-*sn*-*glycero*-3-phospho-l-serine (POPS) (POPC/POPS 8:2, mol:mol) as well as its mixture with POPS and 1-palmitoyl-2-oleoyl-*sn*-*glycero*-3-phosphatidylinositol-(3,4,5)-triphosphate (POPIP3) (POPC/POPS/POPIP3 7:2:1, mol:mol). Again, only the required number of counter-ions in form of sodium and no additional salt concentration was added.

For the multilayer system (Fig. 7) the POPC:POPS (8:2 mol:mol) bilayer was first run separately in the presence of 400 mM Na^+^ or Ca^2+^, respectively. Hereby, the bilayer had a hole with a diameter of around 2 nm produced by a cylindric force constant of 1000 kJ/(mol nm^2^), which only was applied to beads C3A and C3B of POPC and POPS being in a 2 nm environment around a chosen point at *x =* 35 Å and *y =* 35 Å. Then the COOH-OEG-SAM layer was run separately in the presence of 400 mM Na^+^ or Ca^2+^, respectively. Hereby, the *x/y*-dimensions of the box were set to be equivalent to the *x/y*-dimensions of the final frame of the bilayer simulation. Both layers were then added together into a box with the given *x/y*-dimensions and *z* = 450 Å, separated by ~ 12 Å. After solvating and evenly distributing 400 mM Na^+^ or 400 mM Ca^2+^ across the water the system ran for 1 µs with a time step of 1 fs, lateral and normal compressibility of 0 and 3.0 × 10^-4^ bar^−1^, respectively. The cation concentrations were calculated as [salt] = *N_c_* × [water]/*N_w_*, where *N_c_* is the number of cations, [salt] the desired salt concentration, *N_w_* the number of water molecules and [water] = 55.5 M [75]. The respective number of chloride ions was added for neutralization.

#### 5.4.4 AA simulation setup:

Following the CG production simulations AA simulations were performed in case of the individual layers. The conversion of the structure from CG to AA resolution was carried out with the Python tool *cg2at.py* [76]. Thereby the counter-ions were replaced with water. As there is no evidence of a favoured protonation site in case of POPIP3, equal amounts of the three different protonation states were included. Then, several calcium and sodium concentrations with respect to the number of water molecules present in the simulation box, were added, evenly distributed across the water. The cation concentrations were calculated as [salt] = *N_c_* × [water]/*N_w_*, where *N_c_* is the number of cations, [salt] the desired salt concentration, *N_w_* the number of water molecules and [water] = 55.5 M [75]. Finally, the number of chloride ions necessary for neutralization was added.

Besides the non-scaled standard Charmm36 force field [43], two scaled Charmm36 force fields with tuned charges and van der Waals radii were applied for each system. For the scaled ECC-Charmm36 force field, the ECC-parameters, recently expanded to phospholipids [77], were applied to the Charmm36 POPC, POPS, POPIP3 and ion parameters. Additionally, the ECC-parametrization was expanded to the SAM molecules. Therefore, the SAM molecules were treated like ECC-lipids and the carboxyl-group together with the polyethylene-groups considered as the tuneable SAM head-group (see Figure SI 9). The scaling factors of the SAM head-group charges and Lennard-Jones-sigma-parameters were set to be equal to the ECC-lipid scaling factors (*f_q_* = 0.75, *f_σ_* = 0.89). For the scaled ECC2-Charmm36 force field only the van der Waals radii of the ions changed compared to ECC-Charmm36. Here, in a previous study for Charmm36 developed ECC-ion-parameters were applied [45]. All ECC and ECC2 ion parameters are given in Table SI 5. Table SI 7 and SI 8 summarize the composition of all simulated AA SAM and AA bilayer systems.

The free energy landscapes were received by applying the Boltzmann equation. Firstly, *N/N_0_* was calculated using the number density profiles and calculating *N_0_* as average of number density above 40 Å away from the surface. Then, the free energy ΔG was calculated using following equation:

(3) $$\Delta G=-RT\times In\left(N/N_{0}\right)$$

where *R* is the gas constant and *T* the temperature (here 308 K).

## CRediT authorship contribution statement

**Laura H. John:** Conceptualization, Investigation, Methodology, Formal analysis, Writing - original draft. **Gail M. Preston:** Funding acquisition, Writing - review & editing. **Mark S.P. Sansom:** Conceptualization, Funding acquisition, Formal analysis, Writing - original draft. **Luke A. Clifton:** Conceptualization, Investigation, Methodology, Formal analysis, Writing - original draft, Funding acquisition.

## Declaration of Competing Interest

The authors declare that they have no known competing financial interests or personal relationships that could have appeared to influence the work reported in this paper.
