# Supplementary material for: Large scale model lipid membrane movement induced by a cation switch
Source: J Colloid Interface Sci. 2021 Aug 15;596:297–311. doi: 10.1016/j.jcis.2021.03.078 (PMC8109235; doi:10.1016/j.jcis.2021.03.078)
Supplement: Supplementary data 1 [file mmc1.docx]

**Supporting Information**

Large Scale Model Lipid Membrane Movement Induced by a Cation Switch

Laura H. John^1,2^, Gail M. Preston^3^, Mark S. P. Sansom^1^ and Luke A. Clifton^2^*

1. Department of Biochemistry, University of Oxford, South Parks Road, Oxford OX1 3QU, UK
2. ISIS Pulsed Neutron and Muon Source, Science and Technology Facilities Council, Rutherford Appleton Laboratory, Harwell Science and Innovation Campus, Didcot, Oxfordshire, OX11 OQX, UK.
3. Department of Plant Sciences, University of Oxford, South Parks Road, Oxford OX1 3RB, UK

Email: [luke.clifton@stfc.ac.uk](mailto:luke.clifton@stfc.ac.uk)

**Section 1: Neutron Reflectometry**

**1.1 Chemicals**


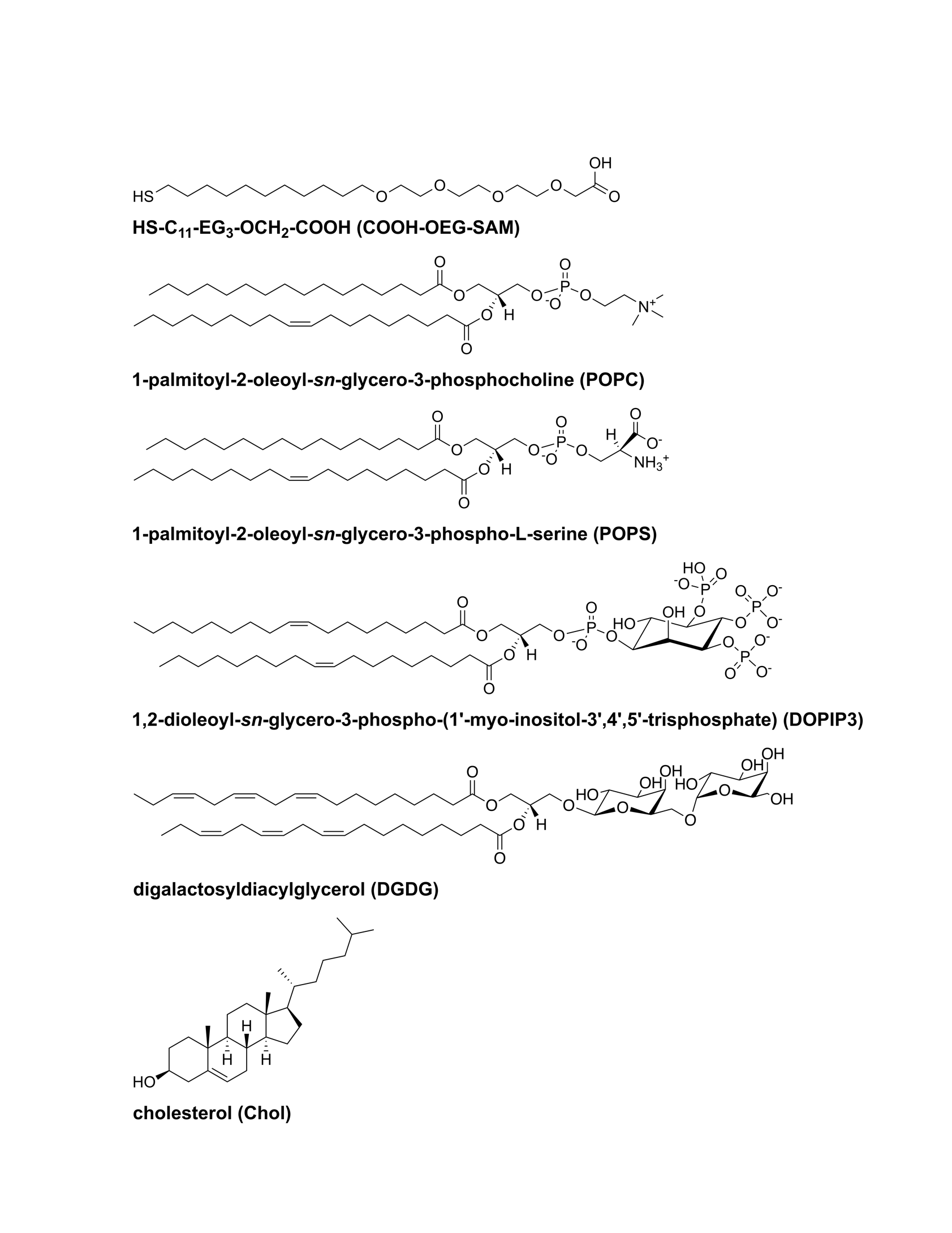


**Figure SI 1 | Chemical Structures of SAM-molecules and Lipid Components:** Structures were produced with ChemDraw.

**1.2 SLD and Reflectometry Profiles**


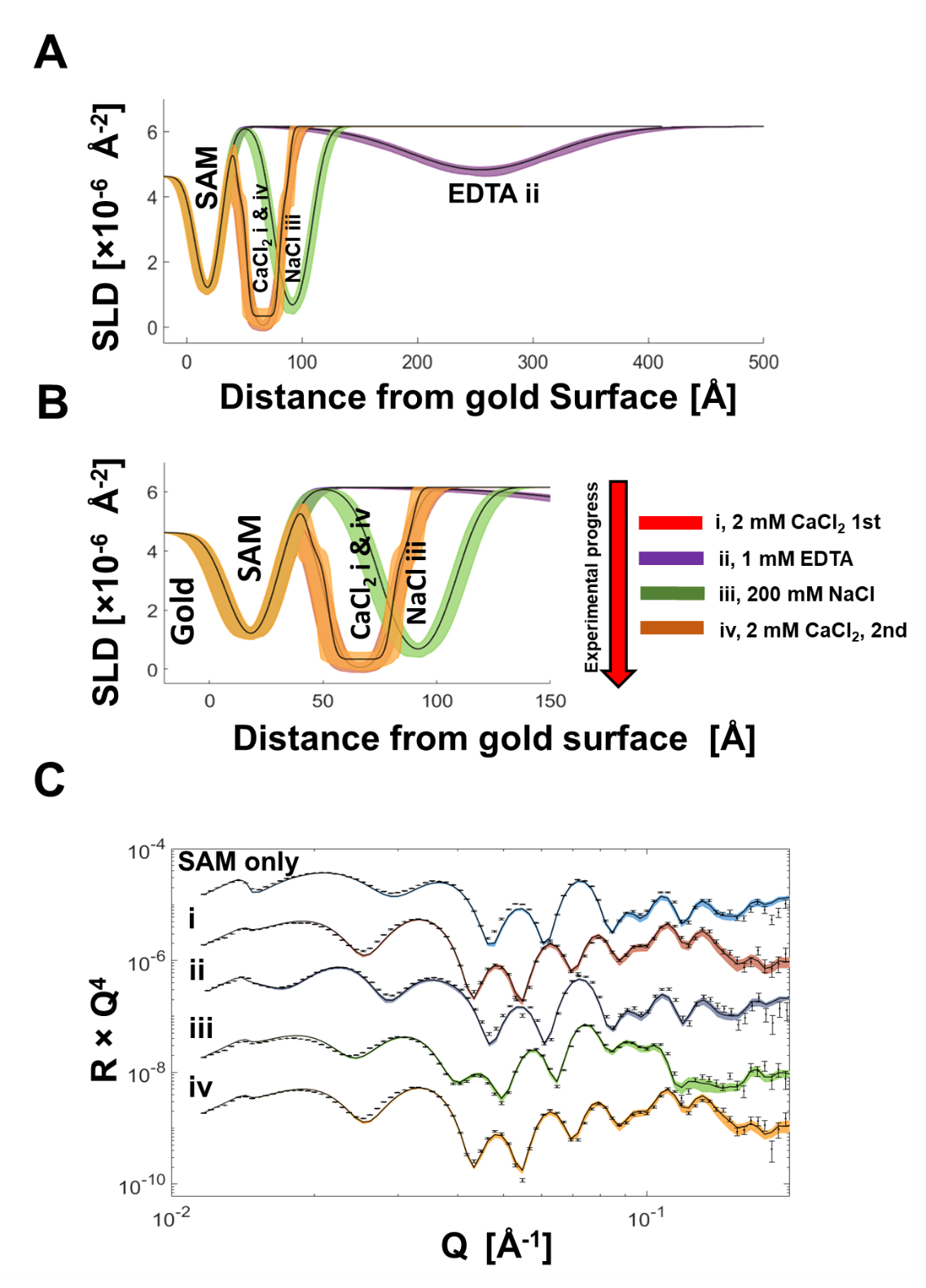


**Figure SI 2 |** Scattering length density (SLD) profiles over two length scale ranges **(A, B),** the corresponding neutron reflectometry profiles and model data fits **(C)** for a POPC bilayer adjacent to a COOH-OEG-SAM coated gold surface at different solution salt conditions. These were, in running order, an initial measurement in 2 mM CaCl_2_ (**i**, red), then 1 mM EDTA (**ii**, purple) followed by 200 mM NaCl (**iii**, green) and finally a return to 2 mM CaCl_2_ (**iv**, orange). The process was fully reversible, so the results in presence of CaCl_2_ before and after the different treatments overlay (i is covered by iv). The reflectivity data and model data fit for the initial COOH-OEG-SAM coated surface without the membrane are shown in blue **(C)**.


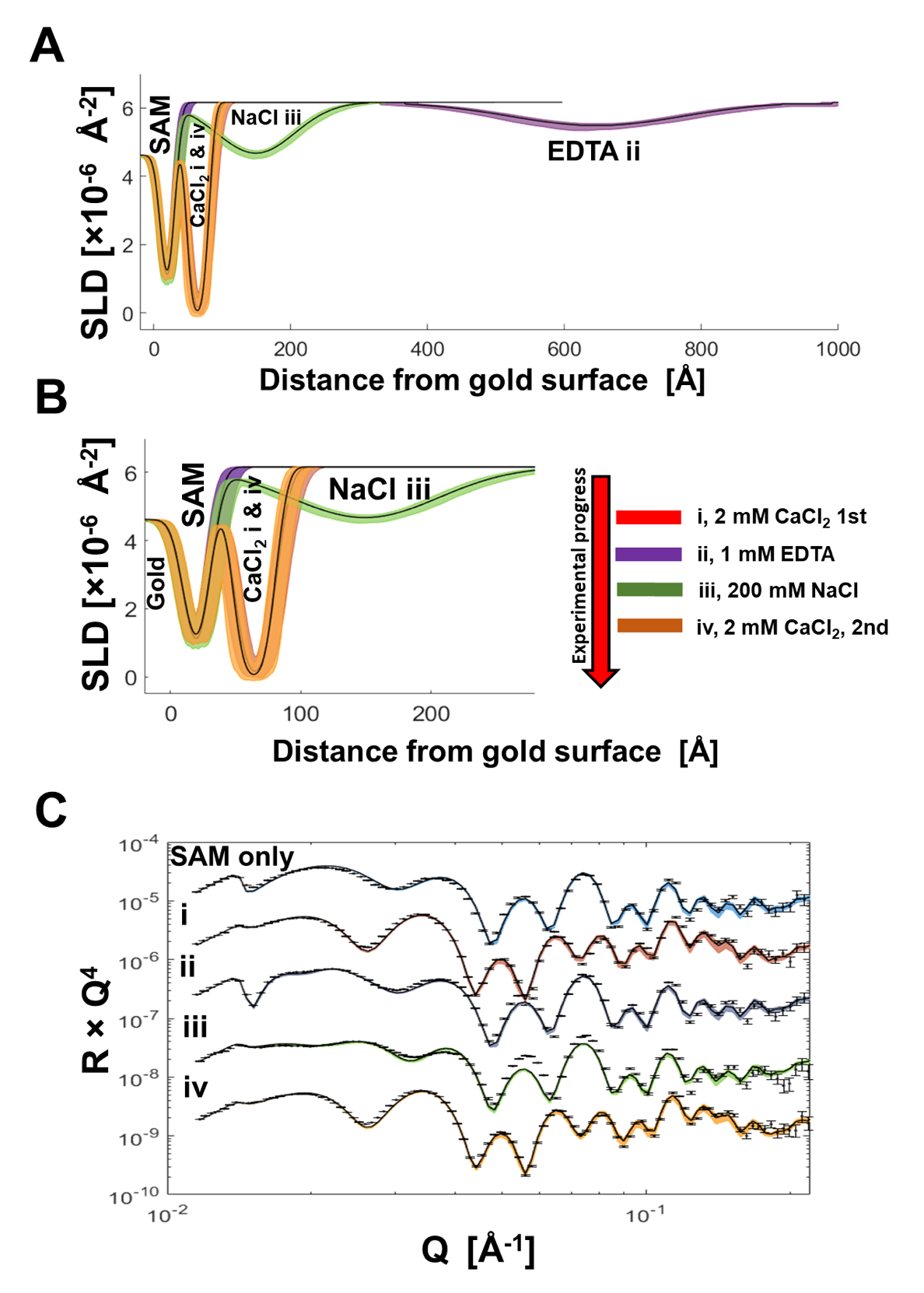


**Figure SI 3 |** Scattering length density (SLD) profiles over two length scale ranges **(A, B)**, the neutron reflectometry profiles and model data fits used to obtain these **(B)** for a POPC:POPS (8:2 mol/mol) bilayer adjacent to a COOH-OEG-SAM grafted to a gold surface under differing solution salt conditions. These were, in running order, an initial measurement in 2 mM CaCl_2_ (**i**, red), then 1mM EDTA (**ii**, purple) followed by 200 mM NaCl (**iii**, green) and finally a return to 2 mM CaCl_2_ (**iv**, orange). The process was fully reversible so the results in CaCl_2_ overlay (**i** is covered by **iv**). The NR data for the COOH-OEG-SAM coated surface are shown (blue data set in **C**).


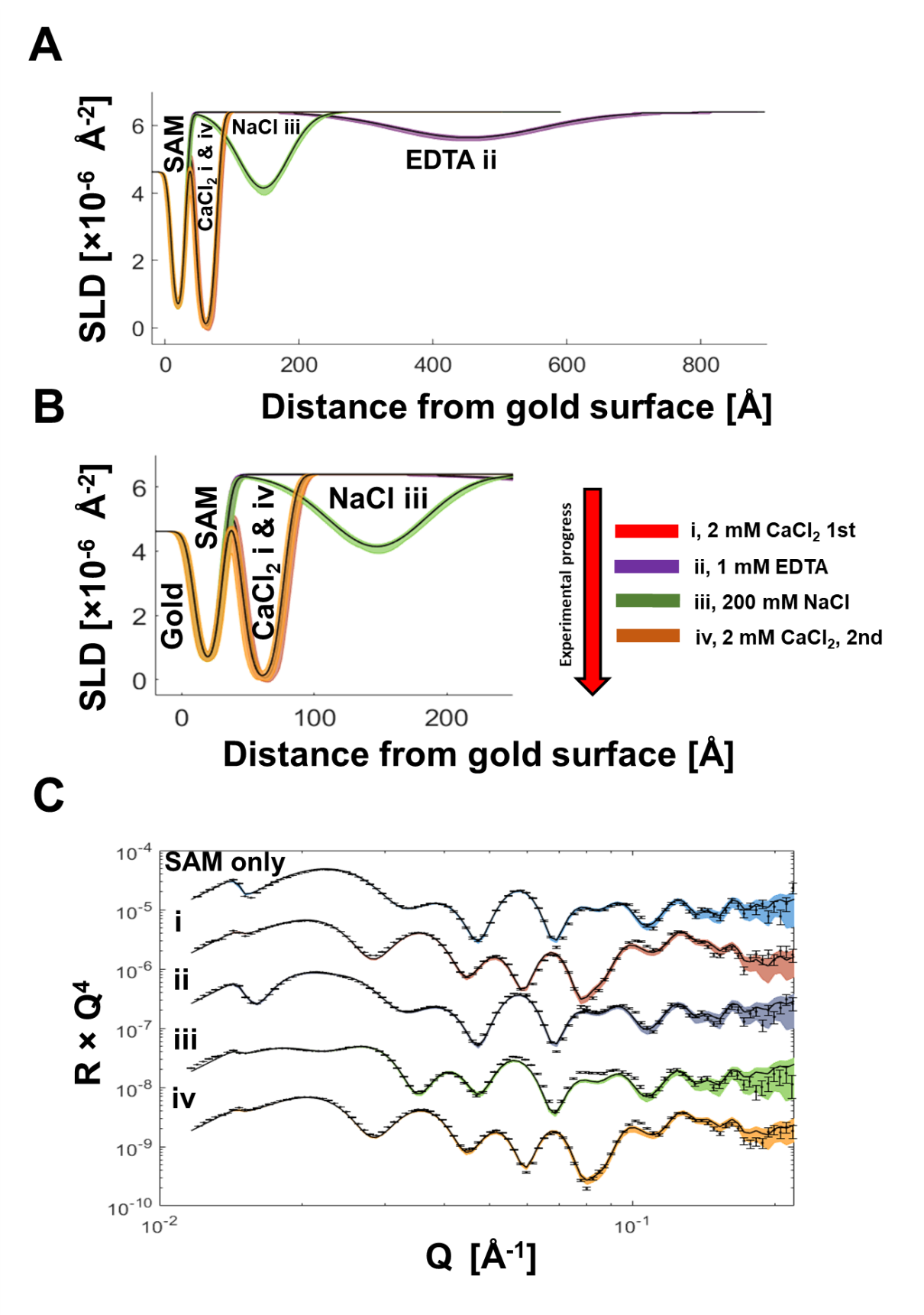


**Figure SI 4 |** Scattering length density (SLD) profiles over two length scale ranges **(A, B)**, the neutron reflectometry profiles and model data fits used to obtain these **(C)** for a POPC:POPS:DOPIP3 (7:2:1 mol:mol) bilayer adjacent to a COOH-OEG-SAM grafted to a gold surface under differing solution salt conditions. These were, in running order, an initial measurement in 2 mM CaCl_2_ (**i**, red), then 1mM EDTA (**ii**, purple) followed by 200 mM NaCl (**iii**, green) and finally a return to 2 mM CaCl_2_ (**iv**, orange). The process was fully reversible so the results in CaCl_2_ overlay (**i** is covered by **iv**). The NR data for the COOH-OEG-SAM coated surface are shown (blue data set in **C**).


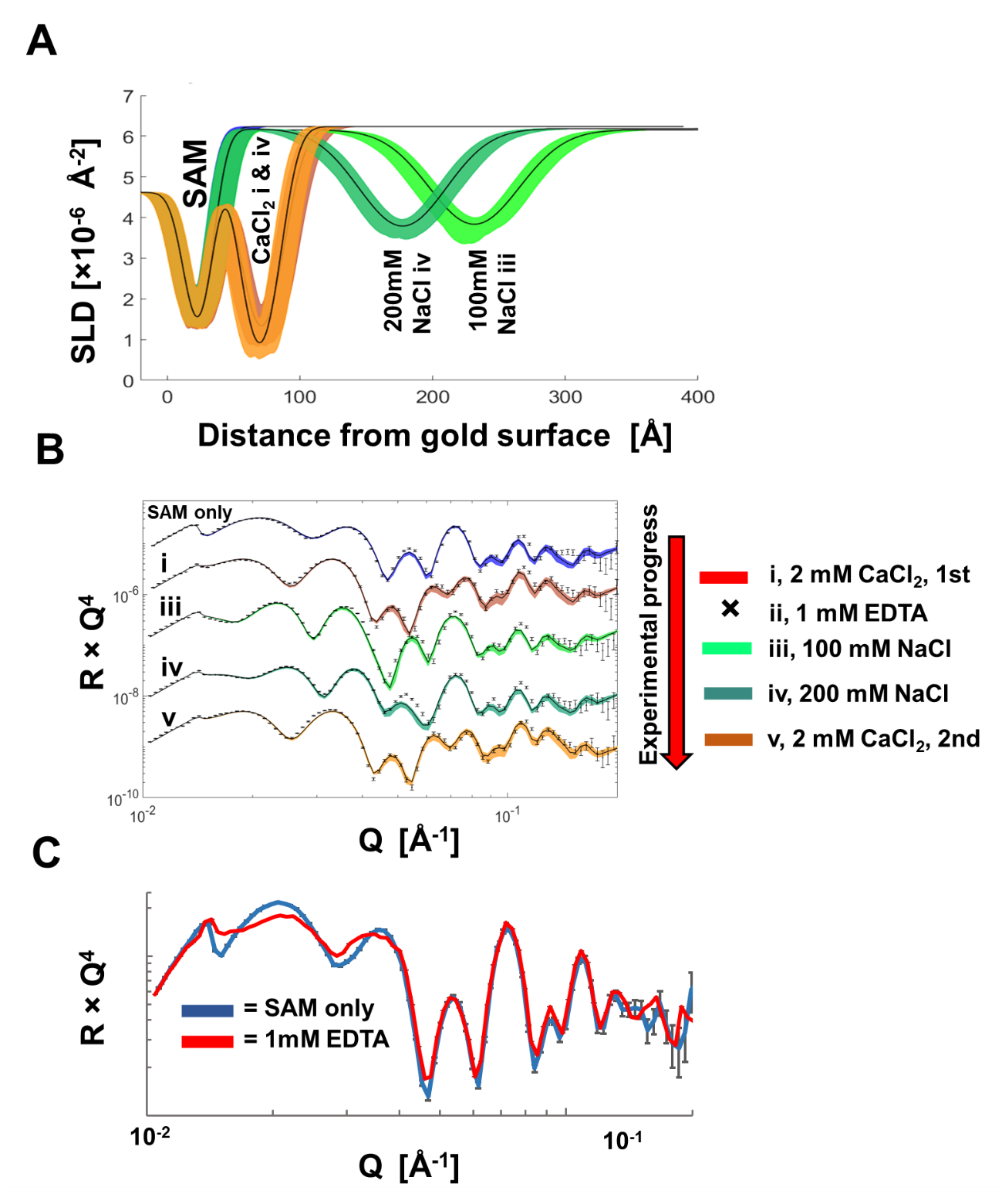


**Figure SI 5 |** Scattering length density (SLD) profiles **(A)**, the experimental reflectivity data and model data fits used to obtain these **(B)** for a POPC:POPS:Chol:DGDG (5:2:2:1 mol:mol) FFB adjacent to a COOH-OEG-SAM coated gold surface at different solution salt conditions. This bilayer was examined at an additional NaCl concentration (100 mM) compared to other samples (Figure S3-S5). A table of the bilayer-to-SAM distance and bilayer roughness for this sample is give in Table S5. A comparison between the data set for the membrane sample in the presence of EDTA compared to COOH-OEG-SAM only data set are shown **(C)**. Though the bilayer was still within the sample cell, as can be seen by this comparison, the EDTA data set could not be successfully analysed probably due to a combination of high bilayer-to-SAM distance, large bilayer roughness and, potentially, positional heterogeneity across the sample surface at high bilayer-to-SAM distances. Parameters for the bilayer-to-SAM distance and bilayer roughness for this sample are given in Table SI 2.

**Table SI 1 | Resolved NR Structural Parameters for Studied FFB Samples:** Parameter ranges as 95% confidence intervals determined from MCMC resampling of the experimental data fits are given in brackets.

| **Sample** | **Area Per Lipid**  **[Å^2^]** | **Initial Bilayer Surface Coverage [%]** | **Final Bilayer Surface Coverage [%]** | **Tails Thickness [Å]** | **Head-group Thickness [Å]** |
| --- | --- | --- | --- | --- | --- |
| **POPC** | 62  (60, 65) | 98  (95, 100) | 95  (89, 100) | 28  (27, 29) | 9  (6, 10) |
| **POPC :POPS**  **8:2** | 63  (60, 65) | 99  (97, 100) | 99  (97, 100) | 28  (27, 29) | 8  (6, 10) |
| **POPC:POPS:DOPIP3**  **7:2:1** | 67  (65, 69) | 99  (97, 100) | 99  (97, 100) | 26  (26, 27) | 7  (6, 9) |
| **POPC:POPS:Chol:DGDG**  **5:2:2:1** | 65  (63, 67) | 100  (98, 100) | 100  (94, 100) | 25  (25, 26) | 9  (6, 11) |


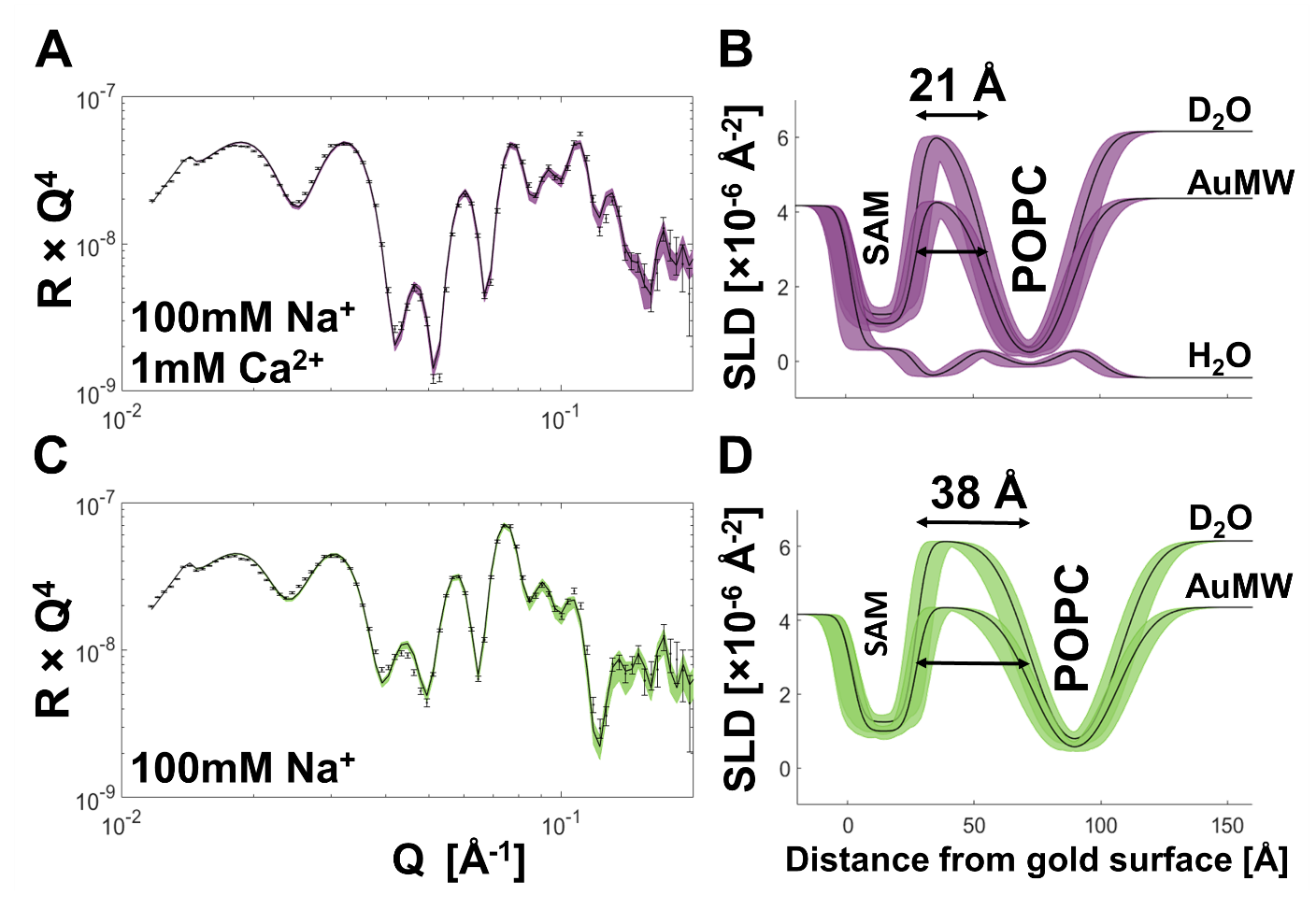


**Figure SI 6 |** Independent repeated neutron reflectometry data examining a POPC membrane adjacent to a COOH-OEG-SAM coated gold surface examined sequentially under two differing solution salt conditions, being 100 mM NaCl / 1 mM CaCl_2_ (purple, **A**) and 100 mM NaCl / 0.5 mM EDTA (green, **C**). Neutron reflectometry data and model data fits from this sample are shown (**A, C**, D_2_O contrast only) and the scattering length density profiles these fits describe are given (**B, D**). The membrane-to-SAM distances under each solution salt condition are given (inset in **B, D**), highlighting the difference in the distance caused with 1mM CaCl_2_ in the presence of 100 mM NaCl. Parameters for the bilayer-to-SAM distance and bilayer roughness for this sample and that given in Figure 3 are given in Table SI 3.

**Table SI 2 | Resolved bilayer-to-SAM distance and bilayer roughness for a POPC:POPS:Chol:DGDG (5:2:2:1 mol:mol) FFB under differing solution salt conditions:** Parameter ranges as 95% confidence intervals determined from MCMC resampling of the experimental data fits are given in brackets

| **Solution Condition** | **Bilayer-to-SAM Distance**  **[Å]** | **Bilayer Roughness**  **[Å]** |
| --- | --- | --- |
| **i,**  **2 mM CaCl_2_** | 15  (13, 17) | 12  (11, 14) |
| **ii,**  **1 mM EDTA** | Data unfittable | Data unfittable |
| **iii,**  **100 mM NaCl** | 175  (172, 179) | 32  (30, 35) |
| **iv,**  **200 mM NaCl** | 121  (118, 125) | 31  (30, 34) |
| **v,**  **2 mM CaCl_2_** | 14  (12, 16) | 10  (8, 11) |

**Table SI 3 | Resolved bilayer-to-SAM distance and bilayer roughness for two sets of independent measurements of POPC bilayer adjacent to COOH-OEG-SAMs in 100 mM NaCl solution with and without 1 mM CaCl_2_:** Parameter ranges as 95% confidence intervals determined from MCMC resampling of the experimental data fits are given in brackets.

| **Sample** | **Parameter** | **100 mM NaCl +**  **1 mM CaCl_2_** | **100 mM NaCl +**  **0.5 mM EDTA** |
| --- | --- | --- | --- |
| **Sample 1**  **(Shown in article**  **Figure 3)** | **Bilayer-to-SAM**  **Distance [Å]** | 24  (22, 26) | 41  (39, 44) |
|  | **Bilayer Roughness**  **[Å]** | 6  (4, 7) | 9  (8, 10) |
| **Sample 2**  **(Shown in SI**  **Figure S6)** | **Bilayer-to-SAM**  **Distance [Å]** | 21  (19, 23) | 38  (37, 40) |
|  | **Bilayer Roughness [Å]** | 8  (6, 9) | 10  (9, 11) |

**Table SI 4 | COOH-OEG-SAM Parameters of each sample determined with NR:** Parameter ranges as 95% confidence intervals determined from MCMC resampling of the experimental data fits are given in brackets.

| **Sample** | **Area Per SAM-molecule**  **[Å^2^]** | **SAM Coverage**  **[%]** | **SAM**  **Thickness**  **[Å]** |
| --- | --- | --- | --- |
| **POPC** | 25  (25, 26) | 99  (97, 100) | 23  (22, 23) |
| **POPC :POPS**  **8:2** | 27  (26, 28) | 99  (98, 100) | 22  (21, 23) |
| **POPC:POPS:DOPIP3**  **7:2:1** | 27  (26, 27) | 99  (97, 100) | 22  (22, 23) |
| **POPC:POPS:Chol:DGDG**  **5:2:2:1** | 27  (26, 28) | 99  (97, 100) | 22  (21, 23) |

**Table SI 5 |** Scattering Length Density (SLD) values for components of the interfacial samples used in the NR studies.

| **Sample**  **Component** | **SLD (ρ)**  **/ 10^-6^ Å^-2^** |
| --- | --- |
| POPC and POPS tails^1^ | -0.3 |
| POPC head groups | 1.98 |
| POPS head groups | 2.92 |
| PIP3 head groups | 2.97 |
| DGDG head groups | 1.31 |
| Cholesterol | 0.157 |
| HS-C_11_-EG_3_-COOH | 0.39 |
| Silicon | 2.07 |
| Gold | 4.6 |
| D_2_O | 6.35 |
| H_2_O | -0.56 |

**Section 2: QCM-D Experiments**


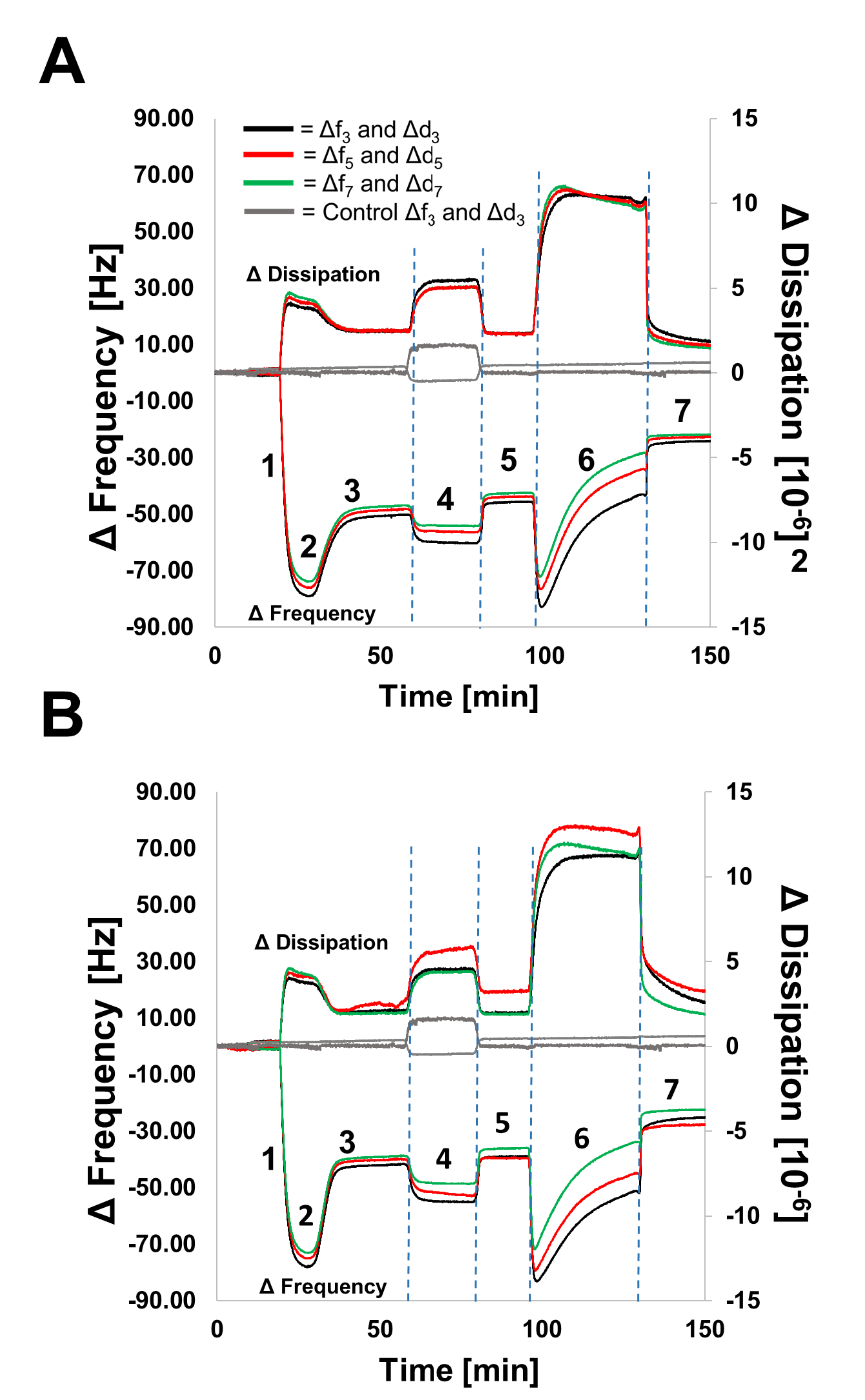


**Figure SI 7 |** Repeated QCM-D measurements showing the change in frequency and dissipation of COOH-OEG-SAM coated gold sensor surfaces during the deposition of a FFB of POPC:POPS (8:2, mol:mol) and subsequent washes with differing solution salt conditions. Only Δ*f* and Δ*d* for the 3^rd^ (black line), 5^th^ (red line) and 7^th^ (green line) overtones are given for clarity in the display of the repeated runs (**A, B**). For the control data set with no membrane present (grey line) only the 3^rd^ overtone is given for comparison. Steps 1-3 detail stages of the FFB fabrication using this technique with the adsorption of vesicles to the sensor surface **(1),** vesicle rupture **(2)** and buffer solution flushing with 20 mM HEPES, 2mM CaCl_2_ pH 7.2 **(3)**. Further steps show washes with 20 mM HEPES pH 7.2 buffer including 200 mM NaCl **(4),** again 2 mM CaCl_2_ **(5),** 1 mM EDTA **(6)** and again 2 mM CaCl_2_ prior to the analysis given in Figures 4 and SI 8 **(7)**.

**
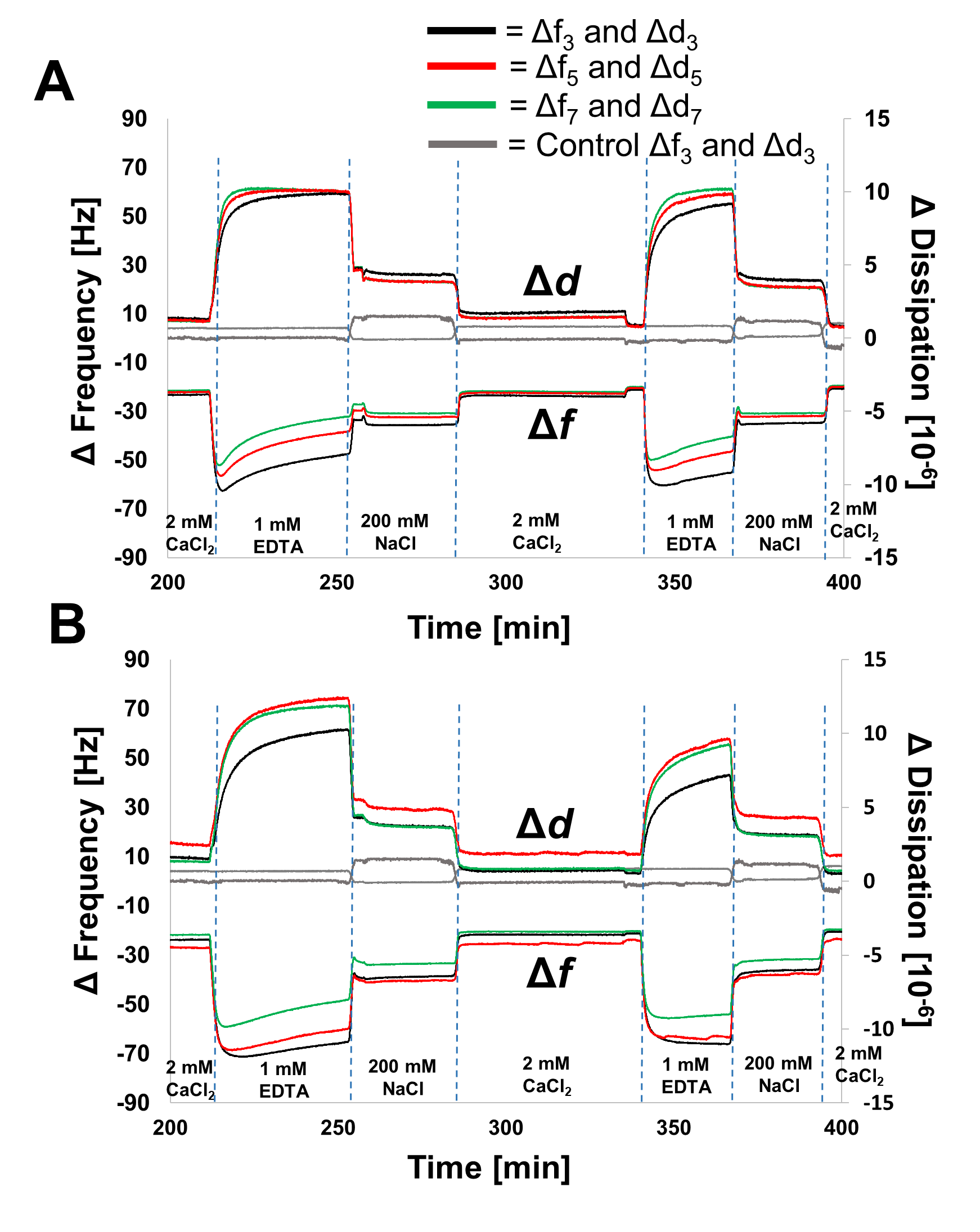
**

**Figure SI 8 |** QCM-D Measurements of a FFB of POPC:POPS (8:2, mol:mol) in absence and presence of CaCl_2_ and NaCl respectively for two independent samples measured simultaneously **(A, B).** The changes in frequency (∆*f*) and dissipation (∆*d*) at different solution salt conditions are given for the 3^rd^ (black), 5^th^ (red) and 7^th^ (green) overtones. For the control data set with no membrane adjacent to the COOH-OEG-SAM only the 3^rd^ overtone is given (grey line). Data from **B** are also given in Figure 4.

**Section 3: MD Simulations**

**Table SI 6 | Ion-Parameters for ECC and ECC2 Force Field Parametrization:** Values for charge (q), Lennard-Jones (LJ) -sigma (σ) and LJ-epsilon (ε) used in respective force field. Unchanged means that the standard Charmm36 value was used.

|  | **ECC-force field**[1]^,^[2]^,^[3] | | | **ECC2-force field**[4] | | |
| --- | --- | --- | --- | --- | --- | --- |
|  | **Ca^2+^** | **Na^+^** | **Cl^-^** | **Ca^2+^** | **Na^+^** | **Cl^-^** |
| ***q*** | 1.5 | 0.75 | -0.75 | 1.5 | 0.75 | -0.75 |
| ***σ* [Å]** | 2.16779 | 2.23717 | 3.17160 | 3.11814 | 2.86869 | 3.17160 |
| ***ε* [kJ/mol]** | unchanged | unchanged | unchanged | 0.0729968 | 0.0303006 | unchanged |


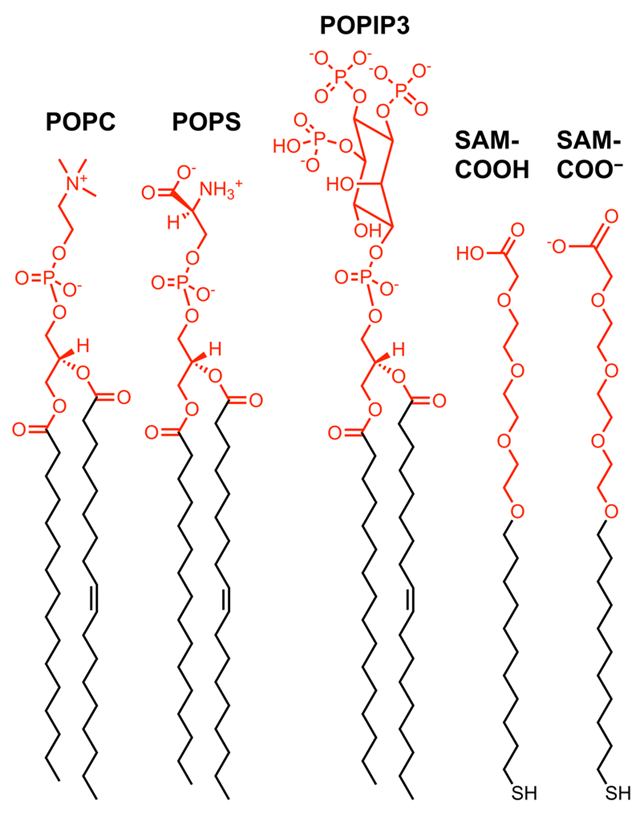


**Figure SI 9 | ECC/ECC2-Parametrization of Lipids and SAM-molecules:** Parts of the molecules considered as headgroup and therefore scaled in their charge and van der Waals radii are shown in red. Unchanged tails of the molecules across the force field parametrizations, meaning standard Charmm36 parameters were used, are shown in black. The scaling factors were: POPC (f_q_ = 0.8, f_σ_ = 0.89), POPS (f_q_ = 0.75, f_σ_ = 0.89), DOPIP3 (f_q_ = 0.75, f_σ_ = 0.89), COOH-OEG-SAM (f_q_ = 0.75, f_σ_ = 0.89), COO^-^-SAM (f_q_ = 0.75, f_σ_ = 0.89). Structures were produced with ChemDraw.

**Table SI 7 | Coarse-grain MD Simulation Parameters**: Parameters for the charged SAM-COO^-^- and uncharged SAM-COOH-molecule. ^*^Bead type of carboxyl group depended on protonation state: for negatively charged, unprotonated carboxyl group SQn and for neutral, protonated carboxyl group SP2. Besides this difference charged and uncharged SAM-molecules were equal in their coarse grained parametrization.

| **Group** | **Name** | **Bead-type** | **Ref.** |
| --- | --- | --- | --- |
| carboxyl | COO | SQn or SP2^*^ | [5]^,^[6] |
| oxydimethylene 1 | PE1 | SN0 | [7]^,^[8] |
| oxydimethylene 2 | PE2 | SN0 | [7]^,^[8] |
| oxydimethylene 3 | PE3 | SN0 | [7]^,^[8] |
| oxydimethylene 4 | PE4 | SN0 | [7]^,^[8] |
| n-butyl 1 | C1B | C1 | [5] |
| n-butyl 2 | C2B | C1 | [5] |
| thioethyl | THI | SC5 | [5]^,^[8]^,^[6] |
|  |  |  |  |
| **Bond** | ***r_0_* [nm]** | ***k_B_* [kJ/mol nm^2^]** | **Ref.** |
| COO-PE1 | 0.29 | 20000 | this work |
| PE1-PE2 | 0.33 | 17000 | [7]^,^[8] |
| PE2-PE3 | 0.33 | 17000 | [7]^,^[8] |
| PE3-PE4 | 0.33 | 17000 | [7]^,^[8] |
| PE4-C1B | 0.39 | 2500 | this work |
| C1B-C2B | 0.47 | 2500 | this work, [5] |
| C2B-THI | 0.45 | 1250 | this work |
|  |  |  |  |

| **Angle** | **𝛳_0_ [°]** | ***k_B_* [kJ/mol rad^2^]** | **Ref.** |
| --- | --- | --- | --- |
| COO-PE1-PE2 | 140 | 70 | this work |
| PE1-PE2-PE3 | 130 | 50 | [7]^,^[8] |
| PE2-PE3-PE4 | 130 | 50 | [7]^,^[8] |
| PE3-PE4-C1B | 140 | 25 | this work |
| PE4-C1B-C2B | 150 | 25 | this work |
| C1B-C2B-THI | 150 | 25 | this work |


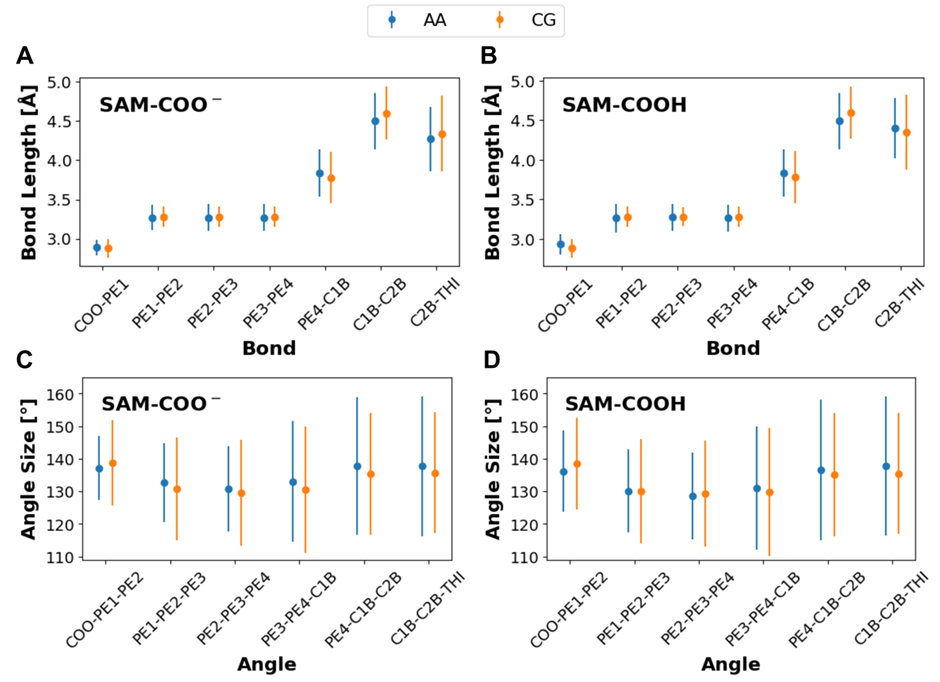


**Figure SI 10 | Mapped Bond Length and Angle Distributions:** Mean and standard deviation of bond length distributions of Martini3-CG (orange) and Charmm36-AA (blue) charged (COO^-^) **(A)** and uncharged (COOH) SAM-molecules **(C)**. Mean and standard deviation of angle distributions of CG (orange) and AA (blue) charged (COO^-^) **(B)** and uncharged (COOH) SAM molecules **(D)**.


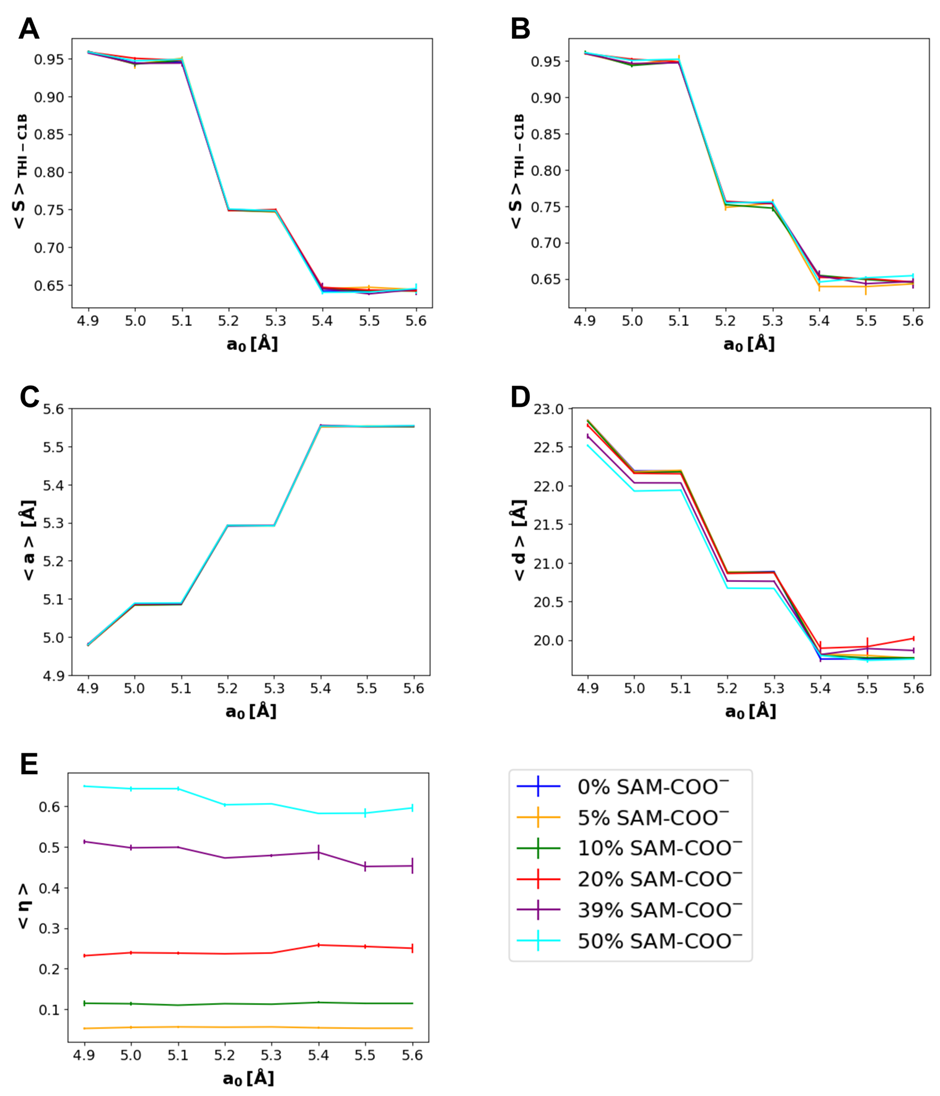


**Figure SI 11 | Characterization of CG COOH-OEG-SAM:** Time and ensemble average of order parameters between thiol-bead and upper CH-chain-bead *<S_THI-C1B_>* of the charged (COO^-^) **(A)** and the uncharged (COOH) **(B)** SAM molecules in dependency of the initial lattice constant *a_0_*. Time and ensemble average of the lattice constant *<a>* **(C)**, the thickness *<d>* **(D)** and the hydrophilicity *<η>* of the SAM **(E)** in dependency of a_0._ All parameters were monitored for SAMs different in charge (see legend for percentage of charged (COO^-^) SAM molecules). The colour code is identical between plots. The surface hydrophilicity was calculated using the GROMACS command SASA with Martini vdW-radii and a probe radius of 0.26 nm. For the calculation of the order parameters, the Martini python script do-order-gmx5.py was used and for the SAM-molecule accordingly edited. As indicated in Figure 5 the thickness was defined as distance between the centre of mass of the thiol-beads (SC5) and the centre of mass of the carboxyl-beads (SQn and SP2).


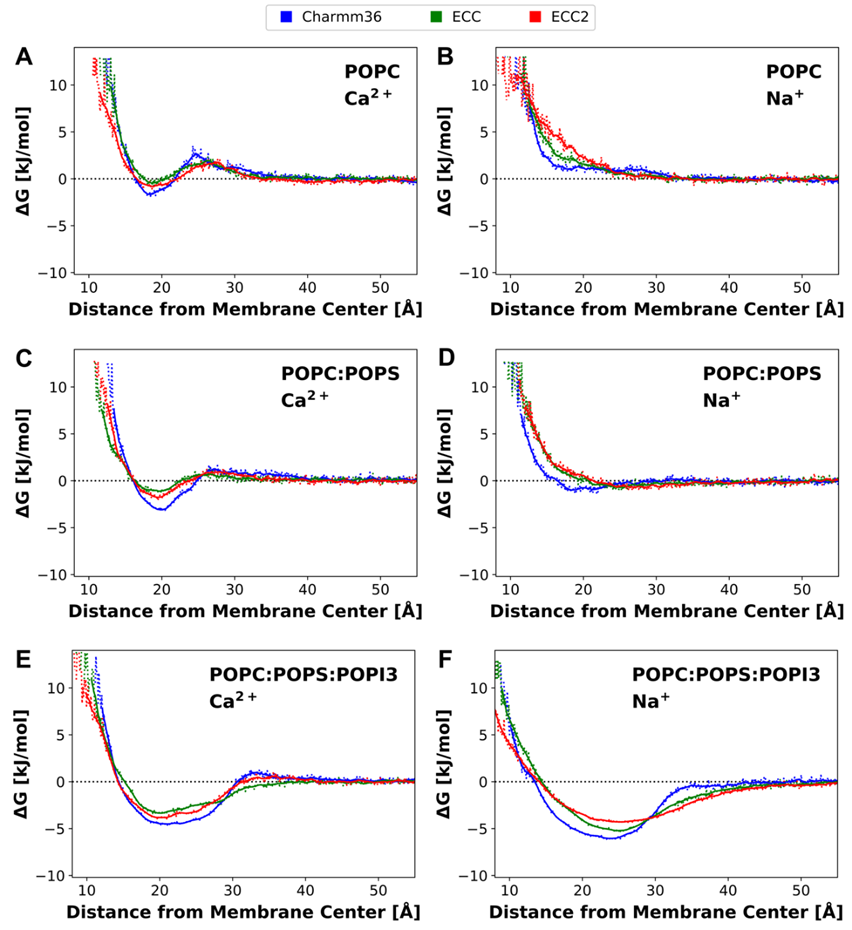


**Figure SI 12 | Free Energy of Cation-Binding at AA Bilayers:** Calculated free energy profiles obtained by applying the Boltzmann equation to simulations in the presence of 400 mM Na^+^ (right column) and 400 mM Ca^2+^ (left column) surrounding different membranes: **(A, B)** POPC, **(C, D)** POPC:POPS (8:2, mol:mol), **(E, F)** POPC:POPS:POPIP3 (7:2:1, mol:mol). Different colours represent three applied Charmm36 force field parameters: Standard (blue), ECC (green) and ECC2 (red). Zero on the *x*-axis is equal to the membrane’s centre of mass. With increasing amount of negatively charged lipids within the membrane the standard Charmm36 force field caused stronger cation-binding compared to the ECC and ECC2 parametrization resulting in deeper minima of the free energy functions **(C-F)**. ECC and ECC2 provided very similar free energy profiles across the different membranes. Therefore, the scaling of the head-group charge seemed to override the effects caused by scaling the head-group Lennard Jones potential.


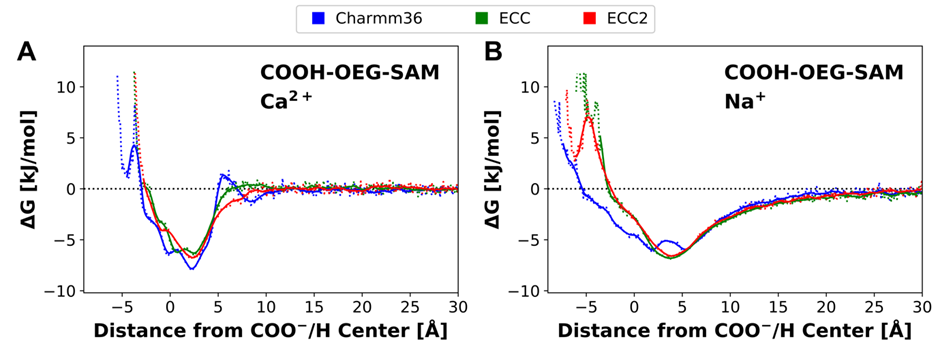


**Figure SI 13 | Free Energy of Cation-Binding at AA COOH-OEG-SAM:** Calculated free energy profiles obtained by applying the Boltzmann equation to simulations in the presence of 400 mM Ca^2+^ **(A)** and 400 mM Na^+^ **(B)** surrounding a COOH-OEG-SAM. Different colours represent three applied Charmm36 force field parameters: Standard (blue), ECC (green) and ECC2 (red). Zero on the x-axis is equal to centre of mass of the SAM carboxyl groups. Ca^2+^-binding was slightly stronger for the standard Charmm36 force field compared to ECC and ECC2 resulting in a deeper minimum of the free energy function **(A)**. In case of Na^+^-binding the standard Charmm36 force field showed a broader free energy profile compared to ECC and ECC2 suggesting a deeper penetration of the cations into the COOH-OEG-SAM **(B)**. The ECC and ECC2 provided very similar free energy profiles for Ca^2+^- and Na^+^-binding.


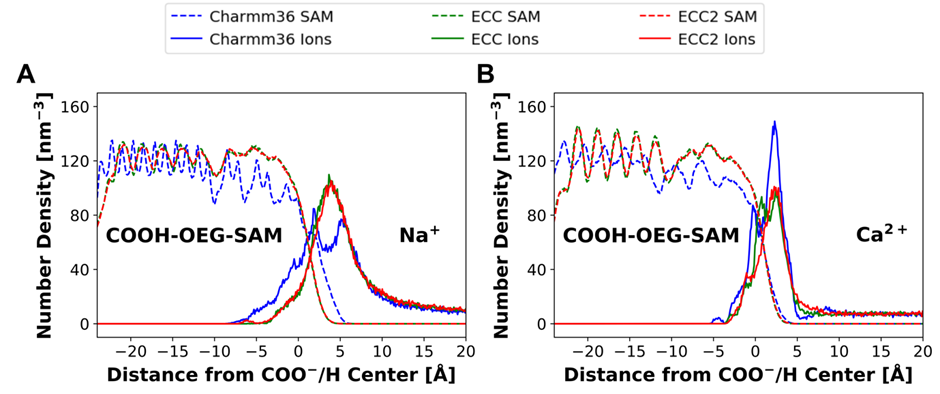


**Figure SI 14 | Partial Densities of AA COOH-OEG-SAM:** Number density profiles of COOH-OEG-SAM (dashed lines) and respective cations (solid lines; **(A)** 400 mM Na^+^, **(B)** 400 mM Ca^2+^) along surface normal. Different colours represent three applied Charmm36 force field parameters: Standard (blue), ECC (green) and ECC2 (red). Zero on the x-axis is equal to centre of mass of the SAM carboxyl groups. In order to visualize the density profiles of the ions, their density was multiplied by 40. Zero on the x-axis is equal to the centre of mass of the SAM carboxyl groups. ECC and ECC2 provided very similar cation number density profiles. By contrast, the standard Charmm36 force field revealed stronger Ca^2+^-binding and deeper penetration of Na^+^-cations resulting in a higher peak for Ca^2+^ and a broader distribution for Na^+^ compared to ECC and ECC2.


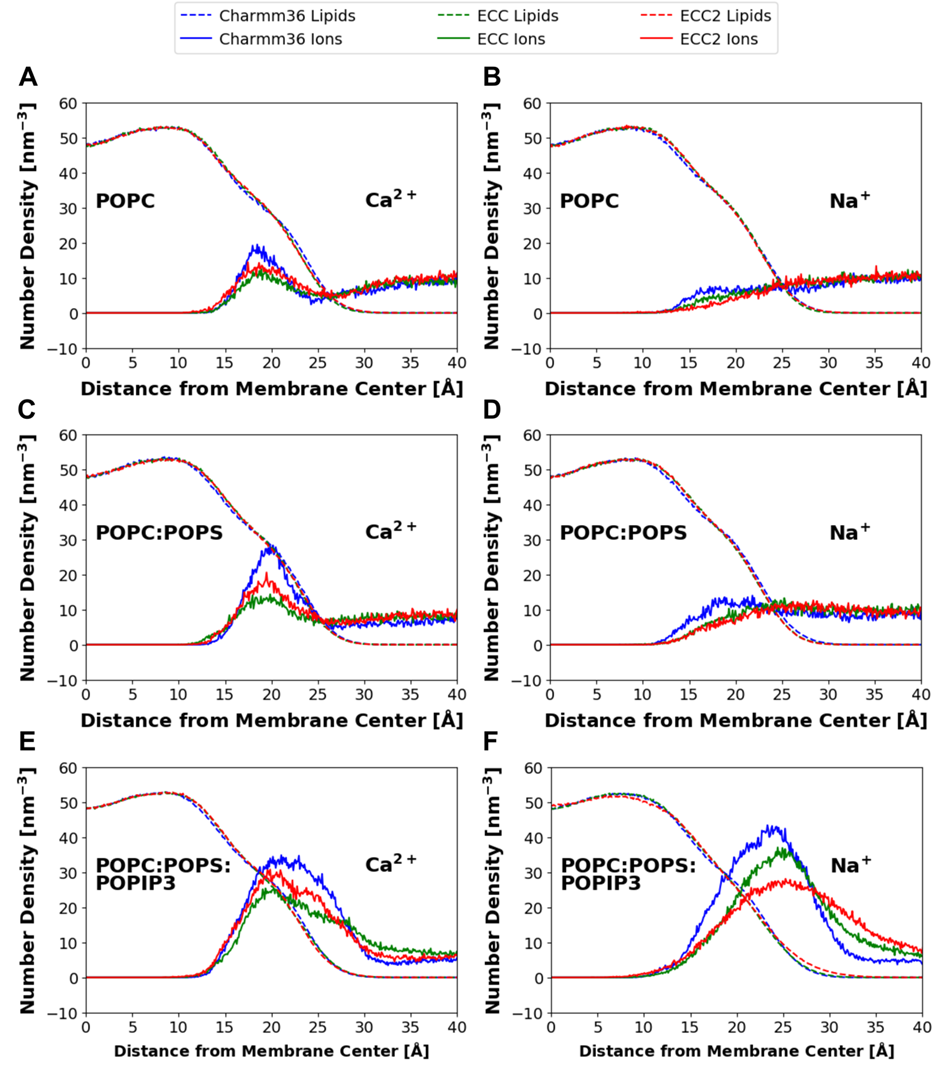


**Figure SI 15 | Partial Densities of AA Membranes:** Number density profiles of lipids (dashed lines; **(A, B)** POPC, **(C, D)** POPC:POPS (8:2, mol:mol ), **(E, F)** POPC:POPS:POPIP3 (7:2:1, mol:mol)) and respective cations (solid lines; **(B, D, F)** Na^+^, **(A, C, E)** Ca^2+^) along surface normal. Different colours represent three applied Charmm36 force field parameters: Standard (blue), ECC (green) and ECC2 (red). In order to visualize the density profiles of the ions, their density was multiplied by 40 and the lipid density by 0.5. Zero on the x-axis is equal to the centre of mass of the membrane.


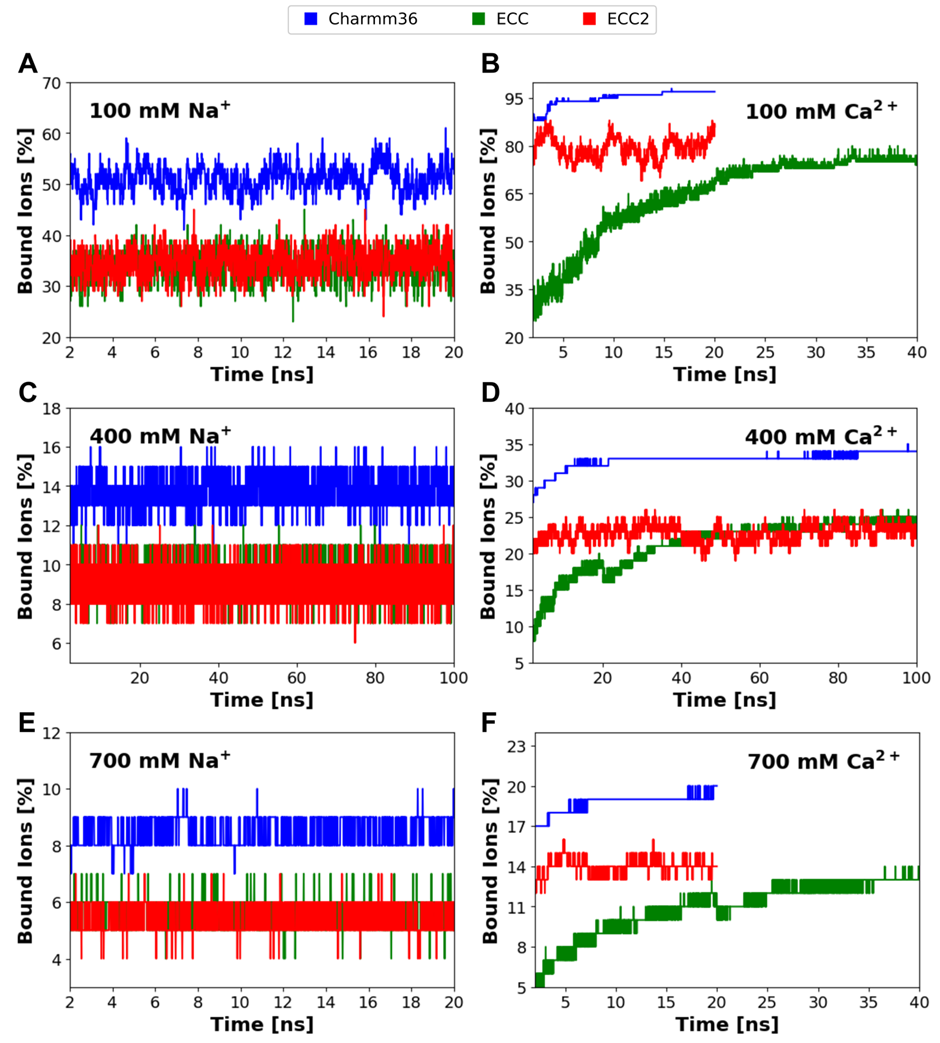


**Figure SI 16 | Bound Cations to AA COOH-OEG-SAM over Time:** Percentage of bound cations over simulation time for three applied Charmm36 force field parameters: Standard (blue), ECC (green) and ECC2 (red). Cation concentration present in simulation box is given in each subplot. The cut-off set to define bound versus unbound states was 3.5 Å. Note the different scaling of axes between subplots. Profiles suggested significant stronger Na^+^ and Ca^2+^ binding to the COOH-OEG-SAM in case of standard Charmm36 compared to ECC and ECC2, whereas ECC and ECC2 seemed to be very similar in terms of ion affinity. The time necessary for obtaining the ion binding equilibrium was longer for ECC in comparison to ECC2. As shown in (**B**), 100 mM Ca^2+^ bind almost completely to the COOH-OEG-SAM in case of standard Charmm36. Therefore, simulations with 400 mM ion concentration, showing an equilibrium between bound and unbound ions, were used for calculating free binding energy profiles. Convergence was ensured by calculating the free binding energy profile over simulation time (see Figure SI 17).


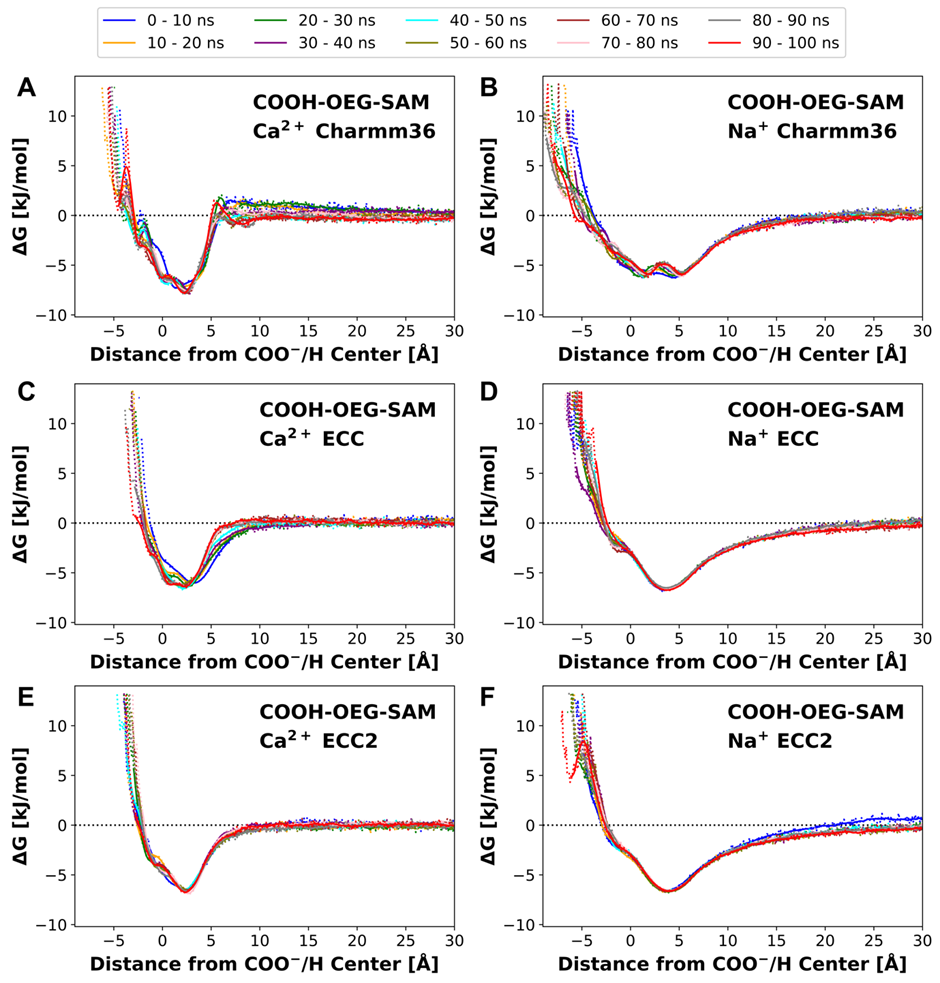


**Figure SI 17 | Free Energy of Cation-Binding at AA COOH-OEG-SAM over Time:** Calculated free energy profiles obtained by applying the Boltzmann equation to simulations in the presence of 400 mM Ca^2+^ and 400 mM Na^+^ surrounding an COOH-OEG-SAM, respectively. Different colours represent 10 ns time periods along the production run (see legend). Three Charmm36 force field parameters were applied: Standard (**A, B**), ECC (**C, D**) and ECC2 (**E, F**)**.** Zero on the x-axis is equal to centre of mass of the SAM carboxyl groups. Convergence was either already during the NPT-equilibration reached or at the latest after 30-40 ns of the production run.


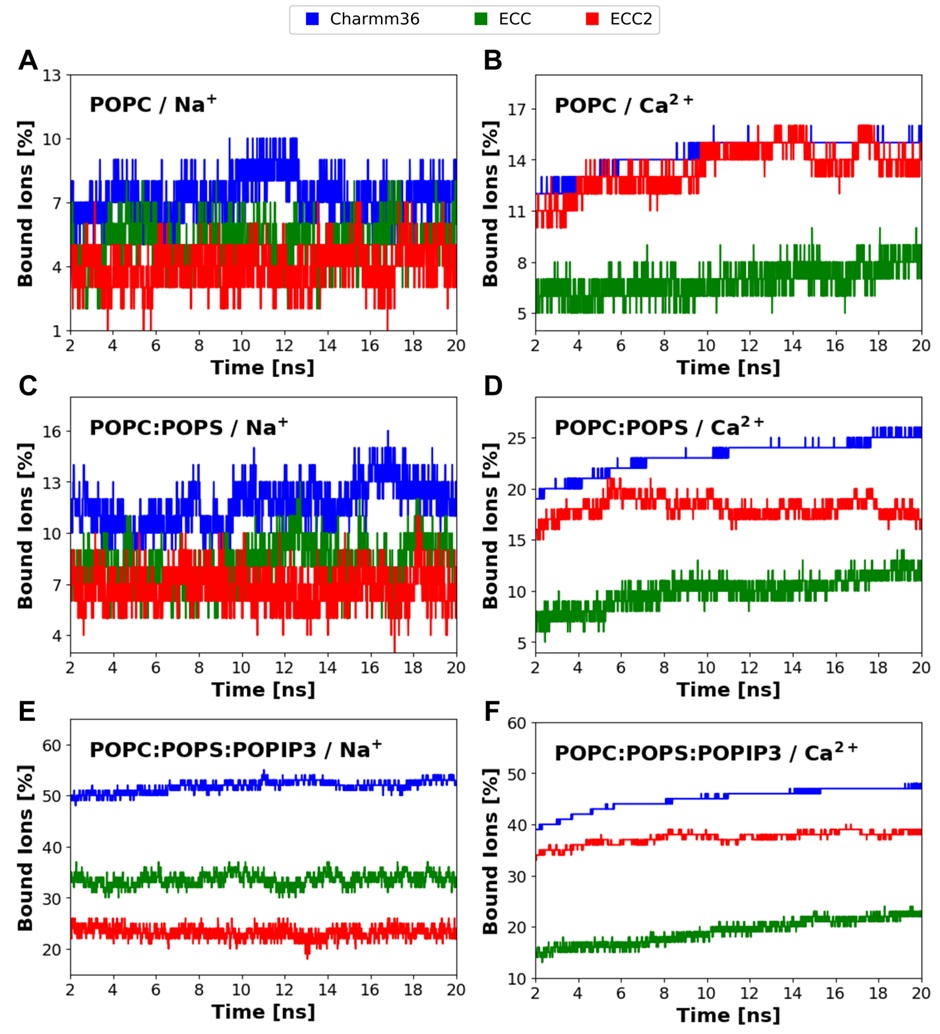


**Figure SI 18 | Bound Cations to AA Bilayers over Time:** Percentage of bound cations over simulation time for three applied Charmm36 force field parameters: Standard (blue), ECC (green) and ECC2 (red). The cation concentration was always 400 mM. The type of cation, as well as the phospholipid composition of the respective bilayer present in the simulation box is given in each subplot. The cut-off set to define bound versus unbound states was 3.5 Å. Note the different scaling of y-axes between subplots.


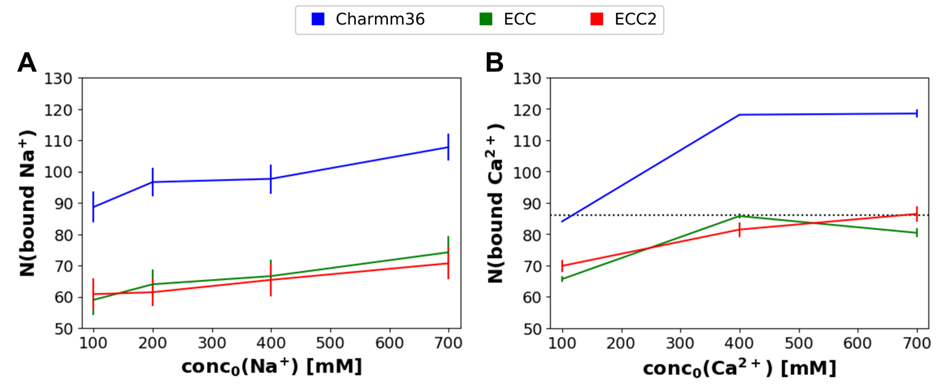


**Figure SI 19 | Number of Bound Cations to AA COOH-OEG-SAM at Equilibrium:** Average and standard deviation of the number of bound Na^+^ **(A)** and Ca^2+^ **(B)** over the last 5 ns of the production simulations for three applied Charmm36 force field parameters: Standard (blue), ECC (green), ECC2 (red). The x-axis is showing the given cation concentration (conc_0_) in the simulation box. The cut-off between cations and COOH-OEG-SAM set to define bound versus unbound states was 3.5 Å. The number of negatively charged SAM-COO^–^-molecules was for all simulations 171. The dotted, black horizontal line in **(B)** represents the number of Ca^2+^ necessary for neutralizing this number of given charges in the COOH-OEG-SAM. For Na^+^, neutralization would need 171 cations, which is above the values given in **(A)** and therefore not shown here.


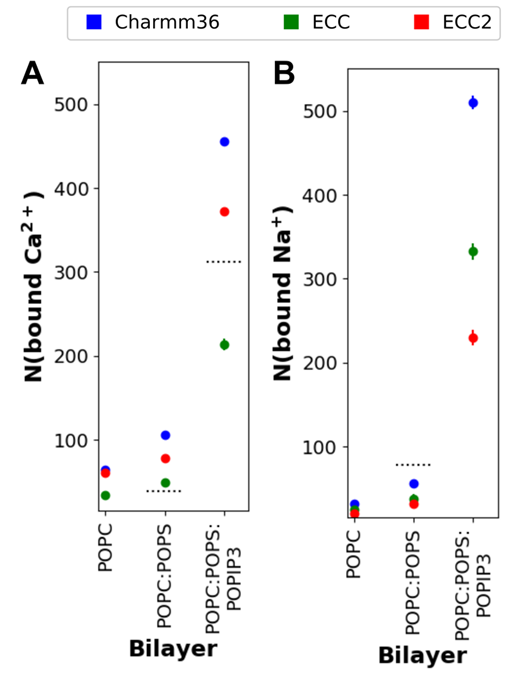


**Figure SI 20 | Number of Bound Cations to AA Bilayers at Equilibrium:** Average and standard deviation of the number of bound Ca^2+^ **(A)** and Na^+^ ions **(B)** over the last 5 ns of the production simulations for three applied Charmm36 force field parameters: Standard (blue), ECC (green), ECC2 (red). The x-axis is showing the given phospholipid composition. The cut-off between cations and lipids set to define bound versus unbound states was 3.5 Å. The standard deviation is mostly too small to be seen. The dotted, black horizontal lines represent the number of Ca^2+^ or Na^+^ ions necessary for neutralizing the number of given charges in the bilayers containing negatively charged POPS and/or POPIP3. For the POPC:POPS:POPIP3 bilayer in the presence of Na^+^, neutralization would need 624 bound Na^+^ cations, which is above the values given in **(B)** and therefore not shown here.

**Table SI 8 | Composition of Simulated AA Bilayer Systems:** The first two rows show the concentration of Ca^2+^ and Na^+^ ions with respect to the number of water molecules. The number of molecules and ions present in the respective system are given in the rows below. The number of POPIP3 lipids includes three equal amounts of the three different protonation states.

|  | **POPC** | | **POPC:POPS**  **8:2** | | **POPC:POPS:POPIP3 7:2:1** | |
| --- | --- | --- | --- | --- | --- | --- |
| **Ca^2+^ [mM]** | 400 | - | 400 | - | 400 | - |
| **Na^+^ [mM]** | - | 400 | - | 400 | - | 400 |
| **POPC** | 392 | 392 | 312 | 312 | 546 | 546 |
| **POPS** | - | - | 78 | 78 | 156 | 156 |
| **POPIP3** | - | - | - | - | 3 x 26 | 3 x 26 |
| **Water** | 57853 | 58264 | 57935 | 58346 | 133273 | 134213 |
| **Neutralizing cations** | - | - | 39 | 78 | 312 | 624 |
| **Additional cations** | 417 | 420 | 378 | 342 | 646 | 343 |
| **Sum of cations** | 417 | 420 | 417 | 420 | 958 | 967 |
| **Cl^-^** | 834 | 420 | 756 | 342 | 1292 | 343 |

**Table SI 9 | Composition of Simulated AA COOH-OEG-SAM Systems:** The first two rows show the concentration of Ca^2+^ and Na^+^ ions with respect to the number of water molecules. The number of molecules and ions present in the respective system are given in the rows below.

|  | **COOH-OEG-SAM (39% Charged)** | | | | | |
| --- | --- | --- | --- | --- | --- | --- |
| **Ca^2+^ [mM]** | 700 | - | 400 | - | 100 | - |
| **Na^+^ [mM]** | - | 700 | - | 400 |  | 100 |
| **SAM-COO^-^** | 171 | 171 | 171 | 171 | 171 | 171 |
| **SAM-COOH** | 269 | 269 | 269 | 269 | 260 | 269 |
| **Water** | 94453 | 46190 | 46961 | 94221 | 47729 | 94905 |
| **Neutralizing cations** | 171 | 86 | 86 | 171 | 86 | 171 |
| **Additional cations** | 1020 | 513 | 256 | 513 | - | - |
| **Sum of cations** | 1191 | 599 | 342 | 684 | 86 | 171 |
| **Cl^-^** | 1020 | 1027 | 513 | 513 | 1 | - |

**Bibliography**

[1] I. Leontyev, A. Stuchebrukhov, Accounting for electronic polarization in non-polarizable force fields, Phys. Chem. Chem. Phys. 13 (2011) 2613. https://doi.org/10.1039/c0cp01971b.

[2] J. Melcr, H. Martinez-Seara, R. Nencini, J. Kolafa, P. Jungwirth, O.H.S. Ollila, Accurate Binding of Sodium and Calcium to a POPC Bilayer by Effective Inclusion of Electronic Polarization, J. Phys. Chem. B. 122 (2018) 4546–4557. https://doi.org/10.1021/acs.jpcb.7b12510.

[3] J. Melcr, T.M. Ferreira, P. Jungwirth, O.H.S. Ollila, Improved Cation Binding to Lipid Bilayers with Negatively Charged POPS by Effective Inclusion of Electronic Polarization, J. Chem. Theory Comput. 16 (2019) 738–748.

[4] A. Nikitin, G. Del Frate, Development of Nonbonded Models for Metal Cations Using the Electronic Continuum Correction, J. Comput. Chem. 40 (2019) 2464–2472. https://doi.org/10.1002/jcc.26021.

[5] S.J. Marrink, H.J. Risselada, S. Yefimov, D.P. Tieleman, A.H. De Vries, The MARTINI force field: Coarse grained model for biomolecular simulations, J. Phys. Chem. B. 111 (2007) 7812–7824. https://doi.org/10.1021/jp071097f.

[6] Martini3 Beta, (2020). http://cgmartini.nl/index.php/martini3beta (accessed July 21, 2020).

[7] H. Lee, A.H. de Vries, S.-J. Marrink, R.W. Pastor, A coarse-grained model for polyethylene oxide and polyethylene glycol: conformation and hydrodynamics, J. Phys. Chem. B. 113 (2009) 13186–13194.

[8] E. Schulze, M. Stein, Simulation of Mixed Self-Assembled Monolayers on Gold: Effect of Terminal Alkyl Anchor Chain and Monolayer Composition, J. Phys. Chem. B. 122 (2018) 7699–7710. https://doi.org/10.1021/acs.jpcb.8b05075.
